# A Mobile App (mHeart) to Detect Medication Nonadherence in the Heart Transplant Population: Validation Study

**DOI:** 10.2196/15957

**Published:** 2020-02-04

**Authors:** Mar Gomis-Pastor, Eulalia Roig, Sonia Mirabet, Jan T De Pourcq, Irene Conejo, Anna Feliu, Vicens Brossa, Laura Lopez, Andreu Ferrero-Gregori, Anna Barata, M Antonia Mangues

**Affiliations:** 1 Pharmacy Department Hospital de la Santa Creu i Sant Pau Barcelona Spain; 2 Heart Failure and Heart Transplant Unit Cardiology Department Hospital de la Santa Creu i Sant Pau Barcelona, Catalonia Spain; 3 Medicine Department Autonomous University of Barcelona (UAB) Barcelona, Catalonia Spain; 4 Pharmacy Department Hospital de la Santa Creu i Sant Pau Barcelona, Catalonia Spain; 5 Institute of Biomedical Research IIB Sant Pau Biomedical Research Networking Center on Cardiovascular Diseases (CIBERCV) Universitat Autonoma de Barcelona Barcelona, Catalonia Spain; 6 Moffitt Cancer Center Florida, FL United States

**Keywords:** self-report, patient-reported outcome measures, behavioral sciences, treatment adherence and compliance, transplantation, early medical intervention, telemedicine, mobile health, validation studies, patient satisfaction

## Abstract

**Background:**

Medication nonadherence in heart transplant recipients (HTxR) is related to graft loss and death. mHeart is a mobile app that uses electronic patient-reported outcome measures (ePROMs) to identify and manage medication nonadherence in the outpatient heart transplant (HTx) population.

**Objective:**

The study primarily aimed to validate mHeart to measure medication nonadherence in early stage HTxR by assessing the psychometric properties of ePROMs. The secondary aims were to (1) measure patient satisfaction with the mHeart tool and its usability and (2) explore the impact of a theory-based treatment on medication nonadherence rates to determine its scalability to larger research.

**Methods:**

A prospective study was conducted in the outpatient clinic of a tertiary hospital. All consecutive early stage HTxR (<1.5 years from HTx) were included. The ePROM psychometric properties assessed were validity, reliability, responsiveness, interpretability, and burden. ePROMs comprised the 4-item Morisky-Green-Levine questionnaire and an adapted version of the Haynes-Sackett questionnaire. The Simplified Medication Adherence Questionnaire (SMAQ) was also applied on-site. Three consecutive medication nonadherence assessments were performed by a transplant pharmacist. To improve medication nonadherence, theory-based interventions were delivered in a 1-month period. Patient satisfaction was assessed by a semiquantitative Web-based survey at the end of the study.

**Results:**

We included 31 early stage HTxR (age: mean 54 years, SD 12 years), and 71% (22/31) of them were men. The HTxR were taking a mean 13 (SD 4; range 7-18) drugs per day. A total of 42% (13/31) of patients were unaware of the consequences of medication nonadherence, and 39% (12/31) of patients were nonadherent to immunosuppressive treatment. The content validity measure showed excellent levels of expert panel agreement for the Haynes-Sacket (14/14, 100%) and Morisky-Green-Levine (13/14, 93%) questionnaires. SMAQ and Morisky-Green-Levine ePROMs showed similar measurement domains (convergent validity, phi=0.6, *P*<.001), which, as expected, differed from Haynes-Sackett ePROMs (divergent validity, phi=0.3, *P*=.12). Reliability assessment revealed a very strong association between ePROM and on-site PROMs (phi>0.7, *P*<.001). Reproducibility was moderate (Haynes-Sackett κ=0.6, *P*<.002) or poor (Morisky-Green-Levine κ=0.3, *P*=.11) because of unexpected improved medication adherence rates during the test-retest period. According to responsiveness, the theory-based multifaceted intervention program improved medication nonadherence by 16% to 26% (*P*<.05). A burden analysis showed that ePROMs could potentially overcome traditional on-site limitations (eg, automatic recording of ePROM responses in the hospital information system). The mean score for overall patient satisfaction with the mHeart approach was 9 (SD 2; score range: 0-10). All 100% (29/29) of patients surveyed reported that they would recommend the mHeart platform to other HTxR.

**Conclusions:**

ePROMs adhered to the quality standards and successfully identified medication nonadherence in the HTx population, supporting their widespread use. The theory-based intervention program showed a promising improvement in medication adherence rates and produced excellent patient satisfaction and usability scores in HTxR.

## Introduction

### Background

Heart transplant recipients (HTxR) require lifelong immunosuppressive therapy to prevent rejection episodes. The estimated percentage of medication nonadherence to immunosuppressive treatment after heart transplant (HTx) ranges from 15% to 30% [1]. These rates are worrisome as medication nonadherence impairs quality of life, increases health costs, and is a direct cause of graft loss and death after HTx [1-6].

Medication nonadherence in the HTx population is a dynamic behavior influenced by multilevel patient, provider, and health system factors [7]. To improve medication adherence, it is essential to frequently monitor medication nonadherence and identify modifiable risk factors for medication nonadherence, such as high therapeutic complexity, weak professional-patient relationship, and lack of patient motivation [4,5,7,8].

Subjective methods to evaluate medication adherence, such as self-reporting, are widely used [9] and well correlated with objective methods (eg, immunosuppressive drug level assay or electronic monitoring systems) [5,10]. Indeed, self-reporting is considered the best method to capture patient experiences and individual risk factors for medication nonadherence, such as patients’ medication beliefs [11]. However, this method involves in-clinic facilities and requires patients to travel to the clinic [5].

Emerging research indicates that patient-reported adherence through mobile devices produces data of similar quality to those provided by traditional in-clinic methods [12]. Therefore, the use of electronic patient-reported measures (ePROMs) to detect nonadherent HTxR could help increase the feasibility of self-reporting and overcome current in-clinic limitations [13,14].

Medication adherence ePROMs could also provide valuable information to care providers to implement early and personalized interventions through mobile health technology. Indeed, internet interventions (ie, “treatments, typically behaviorally based, that are operationalized and transformed for delivery via the Internet”) [13,15-17] show a promising impact on prompting changes in health behaviors such as medication adherence [15,16,18-20].

Behavior-based theories with demonstrated effectiveness in reducing medication nonadherence are recommended to be combined when a new intervention program is designed [21,22]. Moreover, motivational interviewing is a useful tool for professionals to deliver such theory-based interventions in the transplant population [23,24]. Although these behavior change techniques are increasingly used, there is a lack of studies applying them to internet interventions in the HTx population [2,25,26].

### Previous Work

The mHeart medical device is a mobile app that is primarily intended to measure and manage medication adherence in outpatient HTxR. mHeart was designed to use ePROMs to identify medication nonadherence in the home setting and facilitate behavior change interventions to improve medication nonadherence rates. According to the directions for the International Society for Research on Internet Interventions (ISRII) [13], a prerequisite before recommending the widespread use of internet delivery is to demonstrate the accuracy of ePROM scores and their relationship with traditional in-clinic methods [27]. Moreover, the workflow of any new electronic behavior-based intervention designed to manage medication nonadherence should be tested before scaling it to larger research [28].

### Study Objectives

The primary aim of this study was to validate mHeart to measure medication nonadherence in early stage HTxR in the home setting. To do this, we sought to identify the quality of the psychometric properties of the ePROMs reported as being critical in electronic health behavior change instruments [29,30], that is, validity, reliability, responsiveness, interpretability, and burden [31].

The secondary aims were (1) to measure patient satisfaction with mHeart and its usability and (2) to explore the impact of theory-based interventions on medication nonadherence rates among HTxR to determine the hypothetical scalability of the treatment within the context of a larger research study.

## Methods

### Study Design and Setting

This prospective research study was conducted in the ambulatory setting of a Heart Failure and Transplant Unit of a tertiary university hospital from July 15, 2016, to December 1, 2016. The study was approved by the institutional review board of the hospital (IIBSP-MHE-2014-55). The participants were informed of the study purposes, the length of the follow-up, all the procedures, and the research team behind the study. Written informed consent was obtained from all participants.

### Study Reporting Guidelines

The psychometric quality of the ePROMs was based on the Scientific Advisory Committee of the Medical Outcomes Trust (SAC-MOS) [31] and the COnsensus-based Standards for the selection of health Measurement INstruments (COSMIN) consensus guideline [30]. The quality of the results obtained was compared with the International Society for Quality of Life Research (ISOQOL) standards [11].

We followed the European Society for Patient Adherence, COMpliance, and Persistence Medication Adherence Reporting Guideline (EMERGE) [28] recommended criteria for transparent and accurate medication adherence reporting data. The directions for the ISRII [13] and the Consolidated Standards of Reporting Trials of Electronic and Mobile Health Applications and OnLine TeleHealth (CONSORT-EHEALTH) guidelines (section 5) [17] were followed to report the internet-based intervention program. The Theory Coding Scheme (TCS) [32] provided a reliable method to describe the theory underpinning the interventions.

In addition, the Checklist for Reporting Results of Internet E-Surveys [33] was applied to ensure the quality of reporting of the Web-based satisfaction survey.

### Sample

**Figure 1:**
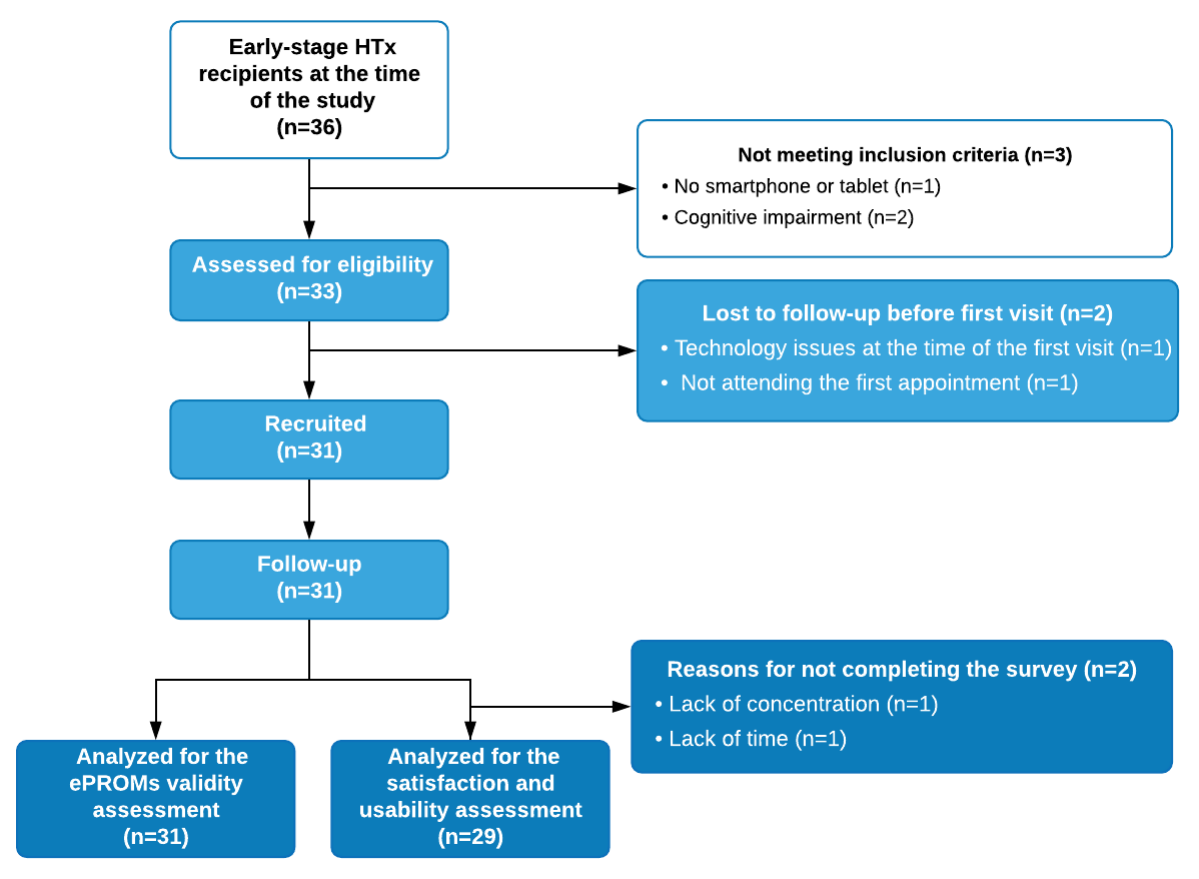
Val-mHeart study patient flowchart. Early-stage: <1.5 years from HTx; ePROMs: electronic patient-reported outcome measures; HTx: heart transplant.

Enrollment was conducted from July 21, 2016, to October 26, 2016, in the Cardiology Outpatient Clinic by transplant physicians during routine in-clinic appointments. All consecutive adult, early stage HTxR (less than 1.5 years from HTx) owning a smartphone and with no cognitive impairment were included. Cognitive impairment was defined as any condition limiting patients’ ability, including memory and thinking skills, to use the mHeart system and complete the questionnaires. No previous computer or smartphone knowledge was required. HTxR did not receive any financial compensation, a phone, or wearables for their participation. The patient flowchart is shown in Figure 1.

### Study Procedures

**Figure 2:**
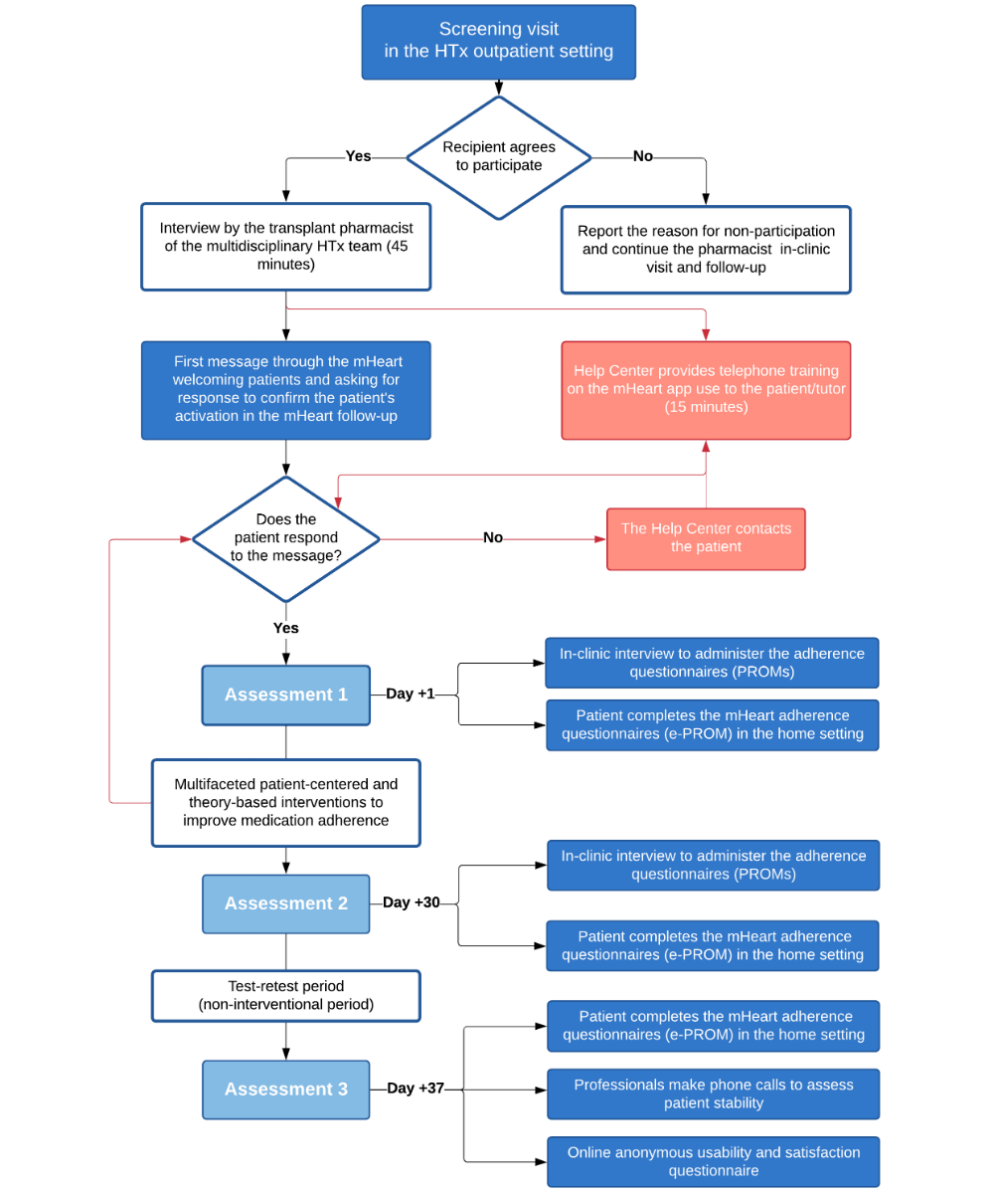
Intervention algorithm summarizing the procedures performed throughout the study period. ePROM: electronic patient-reported outcome measure; HTx: heart transplant; PROMs: patient-reported outcome measures.

The algorithm summarizing the procedures is shown in Figure 2. After signing the informed consent form, all patients were assessed for eligibility (ie, the same day as enrollment by the physicians), and they completed a baseline face-to-face visit with the transplant pharmacist, followed by an initial mHeart training session.

The interview with the pharmacist lasted approximately 45 min. Sociodemographic and clinical data were extracted from patients’ electronic health records. The data were collected in a form provided in Multimedia Appendices 1 and 2. At the end of the visit, the pharmacist registered the new patient’s profile in the mHeart system. Patient access was facilitated by an automated message sent to the patient’s phone with a username and password.

Thereafter, a technical mHeart initial setup was provided by the mHeart Help Center of the private firm developing the technology. This session was conducted by telephone and lasted at least 15 min to enable at-home monitoring, that is, (1) downloading the app from the app store, (2) guiding the first access, and (3) providing training on the functionalities of the mHeart platform. This service was also responsible for query resolution and user assistance throughout the study.

As soon as the HTxR had received training, the transplant pharmacist sent them a welcome message through mHeart, requesting the patients’ response to confirm their activation in the mHeart follow-up. Once the patients had responded to this message, three consecutive assessments were scheduled. The assessment procedures are described below, and these were conducted to measure the validity properties of the ePROMs.

#### Assessment 1

After the baseline visit (ie, on the same day), medication adherence was measured by the pharmacist using in-clinic patient-reported outcome measures (PROMs; Multimedia Appendix 2). No other interventions were performed to manage medication adherence during this in-clinic interview. On the same day, using the mHeart tool, patients were asked to complete the same ePROMs in the home setting.

During the 1-month period between assessments 1 and 2, multifaceted theory-based interventions were provided through mHeart to optimize adherence management [34]. The electronic interventions were interactive, with additional human support from the transplant pharmacist through the mHeart platform. The interventions were individually tailored, based on electronic patient-reported data. Several behavior change techniques [21,22] were used based on those with the strongest evidence base in medication adherence, such as social cognitive theory, the health belief model, transtheoretical model, and self-regulation model. Among others, less often reported but also used are the information-behavior-skill model, self-management theory, behavior modification theory, and problem-solving theory [22]. The techniques were based on Michie’s taxonomy [35] and were delivered using motivational interviewing [23,24] as a common practice pattern to improve posttransplant medication adherence in HTx centers [9]. Interactive elements were also used as digital triggers to counter the law of attrition: alerts, prompts, reminders, notifications, messages, logs, reports, visualizations, and video calls [36,37]. The theoretical framework, the behavior change intervention techniques used, and the intervention workflow are fully described in Multimedia Appendix 3.

#### Assessment 2

Once the intervention program period finished, at least 30 days after assessment 1, the pharmacist conducted an in-clinic interview to perform the second medication adherence PROMs assessment. On the same day, the HTxR were also asked to complete the ePROMs in the home setting.

Thereafter, to allow the test-retest reliability analysis, the patients used mHeart for 7 days without any additional interventions by the pharmacist or contact with the HTx team. At the end of the reproducibility time interval, the patients were telephoned by the pharmacist to confirm clinical and therapeutic stability.

#### Assessment 3

After the test-retest reliability analysis, HTxR were asked to electronically complete the mHeart ePROMs and the satisfaction and usability survey.

### mHeart Features Used During the Study

**Figure 3:**
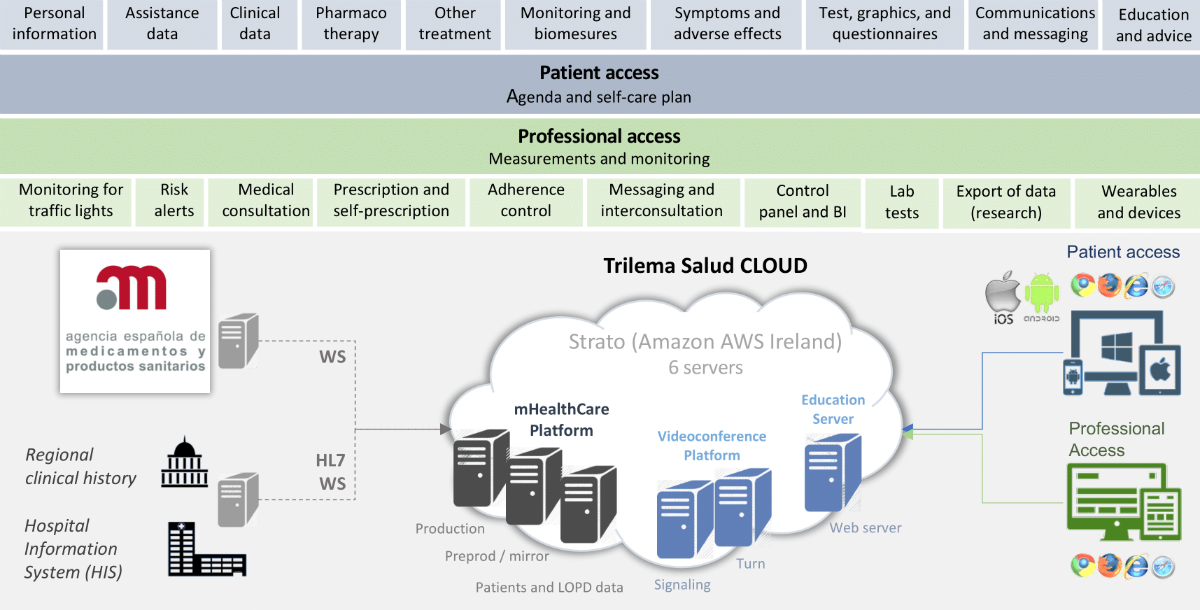
The mHeart functional layer and cloud architecture. AWS: Amazon Web Services; BI: business intelligence; HIS: hospital information system; HL7: high level-7; LOPD: the Spanish Organic Data Protection Law; WS: Web server.

The mHeart medical device is a home-based mobile phone app complemented by a website [38]. From a technical point of view, access to the tool is multiplatform (ie, smartphone, tablet, or computer), and it can be downloaded for free from app stores [39,40]. mHeart is bidirectionally integrated with the hospital information system (HIS) using encrypted data. This integration between the two systems allows mHeart to directly obtain sociodemographic data from the HIS. In addition, mHeart uploads a weekly clinical report to the HIS, including all the data reported by the patients on the platform. The general layout is represented in Figure 3. An in-depth description of the technical specification of the system and the source code are provided in the online Mendely (Dataset) [41]. The version number of the app used was 3.0.2, and the content was frozen during the study.

From a clinical point of view, the mHeart tool was designed to primarily manage medication nonadherence using several features (Table 1). In addition, three of the subfunctionalities of the platform were to (1) resolve patients’ queries about their treatment and health condition, (2) empower patients in terms of self-care, and (3) facilitate professionals’ interventions based on patient-reported outcomes (ie, symptoms and adverse effects of drugs, heart rate, glycemia, weight, and blood pressure). A detailed demonstration of the clinical use of mHeart in HTxR can be found in a video format in Multimedia Appendix 4. More details about functionalities are also provided in the online Mendely (Dataset) [41].

**Table 1:** mHeart platform features related to medication adherence management.

Features	Descriptions
Patient drug intakes	Push text reminds patients of medication intakes on their mobile phone.
	Patients can accept or reject the intakes scheduled. If a patient cancels a dose, they are asked to specify their reason for doing so on a checklist.
	Doses taken versus the total number of doses prescribed can be tracked. A *traffic light* system warns the professional of a decrease in the patient’s weekly adherence. Detailed data are presented for patients and professionals in tables or graphs, including reasons for not taking medication.
Medication adherence ePROMs^a^	The ePROMs included to detect MNA^b^ are the 1-item Haynes-Sackett questionnaire [42,43] adapted to the mHeart platform and the 4-item Morisky-Green-Levine questionnaire [44].
	The professional sets up the frequency of the electronic questionnaire on the patient’s diary.
	Push text alerts on the phone remind the patient to perform the programmed task.
	Test results are shown in tables and graphs to patients and professionals directly from the HIS^c^ or the mHeart platform website.

^a^ePROM: electronic patient-reported outcome measure.

^b^MNA: medication nonadherence.

^c^HIS: hospital information system.

### Measurement Variables

#### Medication Adherence Measures

On the basis of the Ascertaining Barriers to Compliance taxonomy, medication adherence is divided into three phases: initiation, implementation, and persistence [34]. In this study, we focused on assessing the implementation phase of medication nonadherence by using self-reported instruments. Medication nonadherence implementation is defined as “the extent to which a patient’s actual dosing corresponds to the prescribed dosing regimen” (ie, omitting single or consecutive doses, delays in medication intakes, or self-initiated dose changes such as a reduction or increase in dosing). Poor regularity of intakes refers to delays up to 2 hours in the transplant population [45,46]. Medication nonadherence measured by the questionnaires below was defined as any response to items with an answer indicating nonadherence.

**Figure 4:**
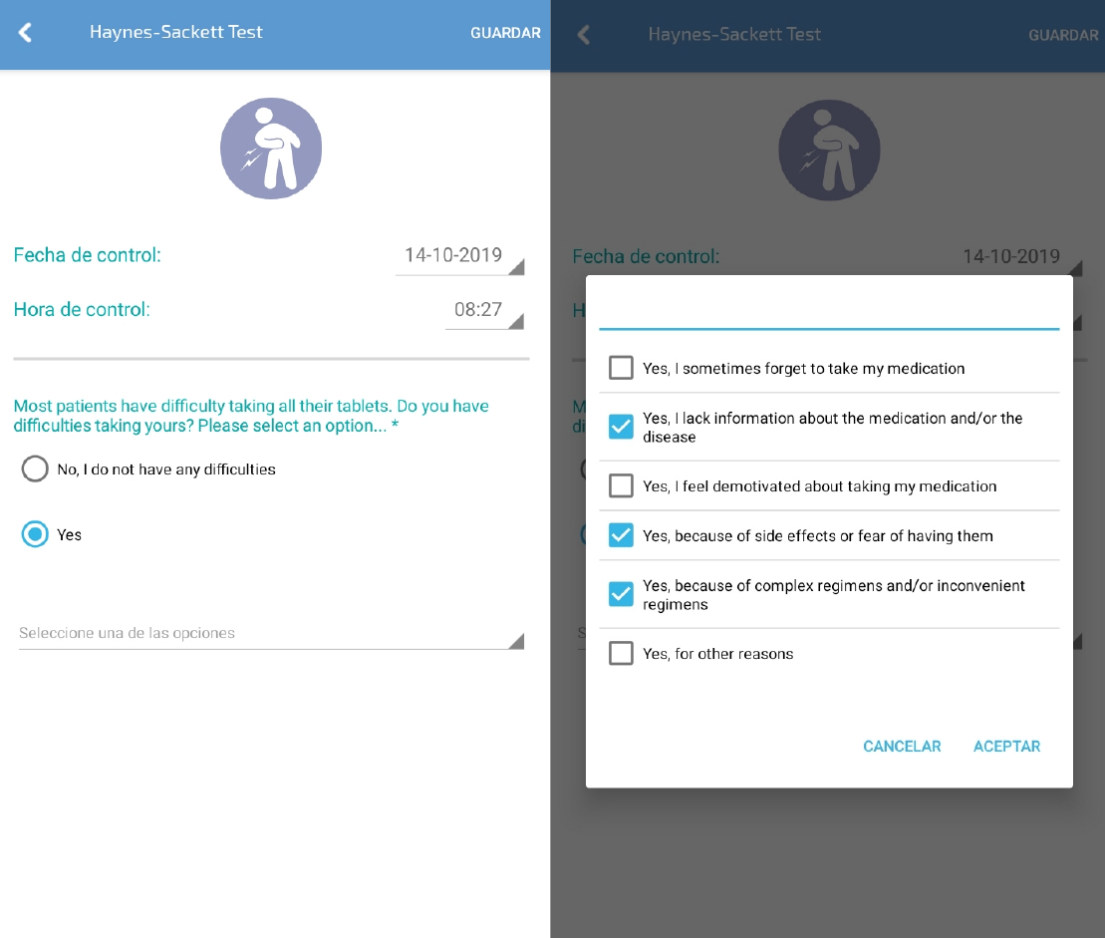
Electronic version of the Haynes-Sackett questionnaire, including 6 additional responses by patients to aid provider understanding of their difficulties with medication adapted for use with the mHeart platform. The score is based on the item 1 response: No (adherent) or Yes (nonadherent).

The ePROM validity study was based on two questionnaires implemented in the mHeart tool. First, the Morisky-Green-Levine questionnaire is a 4-item scale [44] assessing patients’ medication-taking habits. In transferring the questionnaire to an electronic format, we implemented an exact copy of the Spanish validated version [47]. Second, the Haynes-Sackett questionnaire [42,43] is a 1-item scale asking patients whether they have any difficulty with their treatment. In transferring this questionnaire to an electronic format, we implemented the Spanish version [48] and added six multiple-choice responses on patients’ difficulties with medication [49] to improve providers’ understanding of nonadherence (Figure 4 and Multimedia Appendix 5). In both mHeart questionnaires, the items can be answered using Yes or No checkboxes.

For the convergent and discriminant validity assessment, we used the Simplified Medication Adherence Questionnaire (SMAQ) Spanish version as the standard instrument. This questionnaire is a 6-item scale validated in the transplant population receiving immunosuppressive treatment [50]. To identify medication nonadherence risk factors [10], patients were also asked about (1) the knowledge of their regimen, (2) their opinion of the inconvenience of their medication regimens, (3) the importance of the immunosuppressive treatment, and (4) the adverse effects (Multimedia Appendix 3).

#### Patient Satisfaction and Usability

Patient satisfaction with the mHeart intervention program and the usability of the tool were assessed by a Web-based nonvalidated survey created for the study using the *Google Forms* tool. The survey items comprised 8 qualitative and 17 semiquantitative (score range: 0-10) questions (original version in Multimedia Appendix 6). No personal information was collected. Adaptive questioning was used to reduce the complexity of the survey. In addition, all items had a *no response* option, and no blank items were allowed. Respondents were able to review and change their answers before submitting their responses.

The survey was closed to the study participants. The participants were sent an mHeart message by a clinical pharmacist who was different from the transplant pharmacist in charge of the follow-up. The patients had no previous interaction with this provider. The message content comprised an invitation to complete the opinion survey to help the team and developers improve the usability and clinical use of the tool. The patients were assigned a random number from 1 to 31. The survey was voluntary, and no incentives were offered for participation. The patients had 1 week to complete the survey before it was closed to new responses. A reminder was sent to all the HTxR 3 days after the invitation was issued. HTxR accessed the survey through a link uploaded to their mHeart personal profile. Survey completion was permitted by the *Google Form* tool when participants provided their identification number to avoid multiple entries.

The responses and the survey completion rate (ie, the ratio of users who finished the survey/users who agreed to participate) [33] were analyzed in depth. The completion time by participants was not determined.

#### Psychometric Variables to Assess Electronic Patient-Reported Outcome Measure Validity

The psychometric quality of the ePROMs was assessed in terms of validity, reliability, responsiveness, interpretability, and burden [30,31]. The validation measures and methodology are detailed in Multimedia Appendix 7 and briefly described in Table 2.

**Table 2:** Brief description of the validity properties assessed for the mHeart medication adherence electronic patient-reported outcome measures.

Validity properties	Description^a^
Content validity	The interrater agreement among an expert panel was performed to assess the following three content validity aspects. The expert panel comprised 14 health professionals, including 3 nurses, 7 cardiologists, and 4 clinical pharmacists. The suitability of the questionnaires proposed for inclusion in the mHeart app. The discussion was verbal, and voting was by hand. The suitability of the ePROMs^b^ compared with the traditional in-clinic version. After written records were taken, a verbal discussion was held. The suitability of the six medication difficulties added to the electronic version of the Haynes-Sacket questionnaire. After written records were taken, a verbal discussion was held.
Convergent and discriminant validity	Convergent and discriminant validity were assessed using the following aspects: The correlation between the ePROM rates and a standard questionnaire was assessed. The complementarity of the adherence to medication domains of the ePROMs included in the mHeart system was measured.
Reliability (reproducibility)	Reliability and reproducibility were assessed using two methods with different purposes: The equivalent forms reliability method was used to assess the adequate association between the ePROMs scores and the in-clinic scores. With this aim, the PROMs were assessed in the same group of patients and on the same day. The test-retest reliability method was used to assess the stability of the ePROM scores during a short time period (7 days) in clinically stable patients.
Responsiveness (sensitivity to change)	Change over time in medication adherence was measured by the difference in ePROM scores while a theory-based intervention program was performed. A 1-month interval was considered adequate to measure the validity of an indirect smartphone measure [51,52].
Interpretability	Three aspects of the interpretability property were analyzed and discussed: The interpretation of the ePROM scores. The meaningful change detected. The scores obtained versus those published by other authors.
Respondent and administrative burden	Several criteria^a^ were assessed regarding the time, effort, and other criteria of the ePROMs, depending on the respondents’ and administrative points of view.

^a^Full details on validity properties assessed are provided in Multimedia Appendix 7.

^b^ePROM: electronic patient-reported outcome measure.

### Statistical Analyses

#### Descriptive Analysis

Categorical variables are expressed as the number of cases and their percentages, whereas quantitative variables are expressed as mean and SD. Ordinal and quantitative variables not showing normal distribution are expressed as the median and quartiles. McNemar test was used on paired nominal data to determine whether the row and column marginal frequencies were equal. The level of significance was <5% (alpha<.05), bilateral approximation. All analyses were performed using the SPSS version 22.0 (IBM, Armonk, New York) and the R version 3.5.1 (R Project for Statistical Computing, Vienna, Austria).

#### Validity Analysis

The statistical methods used in the validation study are fully detailed in Multimedia Appendix 7. To estimate the interrater agreement measures, an agreement >75% of the expert panel was considered adequate [53]. The one-sample proportion test with continuity correction was applied. Association was measured by the *phi coefficient* (values range from 1 to +1). Phi values above 0.7 are interpreted as showing a very strong association, from 0.4 to 0.69 are interpreted as strong, from 0.3 to 0.39 are interpreted as moderate, from 0.2 to 0.29 are interpreted as weak, and from <0.19 to <0.001 are interpreted as showing no association [53,54]. Agreement was assessed by the *kappa coefficient* (values range from 1 to +1). Kappa values >0.75 are interpreted as strong agreement, from 0.4 to 0.75 indicate moderate agreement, and <0.40 indicate poor agreement [53,55]. In general, values of reliability coefficients >0.80 indicate excellent agreement [56].

#### Sample Size

In this finite population of early stage HTxR, we used a 5 subject-to-variable ratio rule [57]. Therefore, a sample size greater than or equal to 25 participants for a total of 5 items (1-item Haynes-Sackett and 4-item Morisky-Green-Levine questionnaire) was considered the minimum sample required.

To assess validity, reliability (equivalent forms method), responsiveness, interpretability, and burden, we included the entire sample in the analysis. For the test-retest reproducibility study, we included HTxR who remained stable for 7 days [31]. Stability was defined as the absence of need for medication changes or health center consultation and the absence of any symptoms different from those present at the last clinical evaluation.

## Results

### Participant Characteristics

A total of 31 early stage HTxR were included (age: mean 54 years, SD 12 years) and analyzed, and no attrition was observed (Figure 1). In all, 71% (22/31) of participants were men. The mean follow-up was 2.3 (SD 0.9) months. The mean time between HTx and the study was 1.2 years (SD 0.8 years). The patients’ demographic and clinical characteristics are detailed in Multimedia Appendix 8.

At baseline, 71% (22/31) of patients used technologies frequently. Most of the patients reported that mHeart could be *useful* (22/31, 71%) or *very useful* (4/31, 13%). One-third of the patients (9/31, 29%) reported that they needed personal assistance to get started with using the mHeart platform.

### Polypharmacy and Determinants of Medication Nonadherence

Polypharmacy was common; the mean total medication count was 13 (SD 4; range 7-18), exceeding 14 drugs per day in 36% (11/31) of patients. Patients reported a mean number of 6 (SD 4) adverse effects. As many as 61% (19/31) of them reported being self-reliant for medication management.

Medication-related inconvenience was moderate to high (>6 of 10) in 25% (8/31) of HTxR. As many as 74% (23/31) of them believed they were taking excessive medication. The danger of sometimes not taking immunosuppressive drugs was understood by 42% (13/31) of recipients. Furthermore, 32% (10/31) of recipients were unaware of the consequences of completely abandoning antirejection therapy. More details are provided in Multimedia Appendix 9.

### Validity Measures

#### Content Validity

Regarding the adequate representability and relevance of the ePROMs to be included in the mHeart system, the Haynes-Sackett and the Morisky-Green-Levine questionnaires showed excellent agreement (>85%), whereas the SMAQ showed poor agreement (<75%; Table 3).

The suitability of the medication difficulties to support its addition to the Haynes-Sackett electronic version was excellent (>80%). Item agreement is detailed in Table 4.

The overall agreement between the ePROMs and the on-site PROMs was strong for the Haynes-Sackett (κ=0.826, *P*<.001) and for the Morisky-Green-Levine (κ=1, *P*<.001) questionnaires. Item agreement is detailed in Table 5.

**Table 3:** Expert panel interrater agreement on the most suitable questionnaires to measure medication adherence using the mHeart platform, measured by the group consensus method.

Round & adherence electronic patient-reported outcome measure		Agreement^a^, n (%)	*P* value^b^	Inclusion in mHeart
**Round 1**				
	Haynes-Sackett	13 (93)	.11	N/A^c^
	Morisky-Green-Levine	12 (86)	.27	N/A
	SMAQ^d^	10 (71)	.50	N/A
**Round 2**				
	Haynes-Sackett	14 (100)	.03	Included
	Morisky-Green-Levine	13 (93)	.11	Included
	SMAQ	6 (43)	.99	Nonincluded

^a^Percentages of agreement. An agreement >75% of the expert panel was considered adequate.

^b^*P* value was one-sided to test whether *P* was greater than .75 (75%).

^c^N/A: not applicable.

^d^SMAQ: Simplified Medication Adherence Questionnaire validated in Spanish transplant population.

**Table 4:** Expert panel interrater agreement on several criteria for the six reasons for medication nonadherence Haynes-Sackett electronic patient-reported outcome measure, measured by the nominal group consensus method.

Reasons for medication nonadherence	Intuitive^a^, n (%)	Easy^a^, n (%)	Brief^a^, n (%)	Useful^a^, n (%)	Percentage of overall agreement^b^, n (%)	*P* value^c^
I sometimes forget to take my medication	14 (100)	14 (100)	14 (100)	14 (100)	14 (100)	<.001
I lack information on medication and/or the disease	14 (100)	13 (93)	14 (100)	14 (100)	13.8 (98)	<.001
I feel demotivated about taking my medication	12 (86)	11 (79)	14 (100)	13 (93)	12.5 (89)	.01
Because of side effects or fear of having them	13 (93)	13 (93)	14 (100)	13 (93)	13.3 (95)	<.001
Because of complex regimens and/or inconvenient regimens	13 (93)	8 (57)	14 (100)	14 (100)	12.3 (88)	.02
Because of other reasons	12 (86)	14 (100)	13 (93)	12 (86)	12.8 (91)	.004

^a^Item criteria full description: true to the original in-clinic test, useful to evaluate medication adherence construct, intuitive, brief or fast to complete, and easy-to-understand language.

^b^Percentages of agreement. An agreement >75% of the expert panel was considered adequate.

^c^*P* value was one-sided to test whether *P* is greater than .75 (75%).

**Table 5:** Expert panel agreement on item characteristics of electronic patient-reported outcome measures compared with on-site patient-reported outcome measures, measured by the nominal group consensus method.

Patient-reported outcome measure item	True^a^		Useful^a^		Intuitive^a^		Brief^a^		Easy^a^	
	Kappa value	*P* value	Kappa value	*P* value	Kappa value	*P* value	Kappa value	*P* value	Kappa value	*P* value
Item 1 MGL^b^	1	<.001	1	<.001	1	<.001	1	<.001	1	<.001
Item 2 MGL	1	<.001	1	<.001	1	<.001	1	<.001	1	<.001
Item 3 MGL	1	<.001	1	<.001	1	<.001	1	<.001	1	<.001
Item 4 MGL	1	<.001	1	<.001	1	<.001	1	<.001	1	<.001
Item 1 HS^c^	1	<.001	0.6	<.01	0.4	.04	1	<.001	1	<.001

^a^Item characteristics’ full description: true to the original in-clinic test, useful to evaluate medication adherence construct, intuitive, brief or fast to complete, and easy-to-understand language.

^b^MGL: Morisky-Green-Levine 4-item questionnaire.

^c^HS: Haynes-Sackett questionnaire.

#### Convergent and Discriminant Validity

The correlation of adherence to medication domains of the PROMs compared with the SMAQ is shown in Table 6.

**Table 6:** Convergent and discriminant validity assessed by the correlation of medication adherence patient-reported outcome measures with the Simplified Medication Adherence Questionnaire.

Validity property	Adherence to medication PROMs^a^	Electronic		In clinic		Interpretation
		Phi coefficient	*P* value	Phi coefficient	*P* value	
Convergent	Morisky-Green-Levine versus SMAQ^b^	0.6	<.001	0.9	<.001	Strong correlation; measures similar adherence domains
Divergent	Haynes-Sackett versus SMAQ	0.3	.12	0.4	.04	Weak correlation; measures different adherence domains

^a^PROMs: patient-reported outcome measures to assess medication adherence.

^b^SMAQ: Simplified Medication Adherence Questionnaire validated in Spanish transplant population.

#### Reproducibility

The equivalent forms reliability method showed a very strong association between the scores obtained using the ePROMs and on-site PROMs (phi>0.7, *P*<.001; Table 7).

For the test-retest reliability method, all participants remained stable between assessments. Low reproducibility was observed, whereas medication adherence improved during this interval according to both types of ePROM (Table 8).

**Table 7:** Reliability of medication adherence electronic patient-reported outcome measures compared with on-site patient-reported outcome measures using the equivalent forms reliability method.

Adherence to medication PROMs^a^	Phi coefficient	*P* value
HS^b^ overall	0.8	<.001
MGL^c^ overall	0.7	<.001
MGL item 1	0.7	<.001
MGL item 2	0.7	<.001
MGL item 3	0.6	<.001
MGL item 4	1	<.001

^a^PROMs: patient-reported outcome measures to assess medication adherence.

^b^HS: Haynes-Sackett questionnaire.

^c^MGL: Morisky-Green-Levine 4-item questionnaire.

**Table 8:** Test-retest reliability method to measure stability of medication adherence electronic patient-reported outcome measure scores over time.

ePROMs^a^	Assessment 2		Assessment 3		Kappa value	*P* value	Interpretation
	Adherent, n (%)	Nonadherent, n (%)	Adherent, n (%)	Nonadherent, n (%)			
HS^b^	29 (94)	2 (7)	31 (100)	0 (0)	0.6	.002	Moderate stability
MGL^c^	28 (90)	3 (10)	30 (97)	1 (3)	0.3	.11	Poor stability

^a^ePROMs: electronic patient-reported outcome measures to assess medication adherence.

^b^HS: Haynes-Sackett questionnaire.

^c^MGL: Morisky-Green-Levine 4-item questionnaire.

#### Responsiveness or Sensitivity to Change

According to the change in medication adherence over time, similar rates were obtained in assessment 2 between ePROMs and PROMs (Table 9). Details for each item are provided in Multimedia Appendix 10.

#### Interpretability

The ePROM scores showed a nonsignificant underestimation (*P*>.05) of medication nonadherence rates at assessment 1 but not at assessment 2. Almost all the patients were adherent according to the ePROMs at assessment 3. The baseline overall in-clinic medication nonadherence rate was 32% (10/31), as measured by the Morisky-Green-Levine PROMs. According to the SMAQ, 39% (12/31) of HTxR were nonadherent to immunosuppressive treatment. The theory-based multifaceted intervention program showed significant (*P*<.05) improvements in medication nonadherence, ranging from 16% to 26%, depending on the questionnaire used (Table 9).

**Table 9:** Medication adherence rates and improvement between study assessments.

Measure		Adherence to medication rates^a^									*P* value		
		Assessment 1^b^		Assessment 2^b^			Assessment 3^b^						
		Adherent, n (%)	Nonadherent, n (%)		Adherent, n (%)	Nonadherent, n (%)		Adherent, n (%)	Nonadherent, n (%)			A1 vs A2	A1 vs A3
**ePROMs^c^**													
	HS^d^	25 (81)	6 (19)		29 (94)	2 (7)		31 (100)	0 (0)			.10	.01
	MGL^e^	25 (81)	6 (19)		28 (90)	3 (10)		30 (97)	1 (3)			.26	.06
**In-clinic PROMs**													
	HS	22 (71)	9 (29)		28 (90)	3 (10)		N/A^f^	N/A			.03	N/A
	MGL	21 (68)	10 (32)		27 (87)	4 (13)		N/A	N/A			.06	N/A
	SMAQ^g^	19 (61)	12 (39)		27 (87)	4 (13)		N/A	N/A			.005	N/A

^a^Medication adherence in the implementation phase is expressed as a binary variable: adherent or nonadherent. The Haynes-Sackett and Morisky-Green-Levine questionnaires measures adherence to overall medication. The Simplified Medication Adherence Questionnaire validated in the Spanish transplant population measures adherence to immunosuppression.

^b^The behavior-based interventional program established by the pharmacist was performed between assessments 1 and 2 (1 month at least). There was a 7-day gap between assessments 2 and 3 to allow the reproducibility test retest study without provider interactions. Only the electronic questionnaires were administered in Assessment 3.

^c^ePROM: electronic patient-reported outcome measures to assess medication adherence.

^d^HS: Haynes-Sackett.

^e^MGL: Morisky-Green-Levine.

^f^N/A: not applicable.

^g^SMAQ: Simplified Medication Adherence Questionnaire validated in the Spanish transplant population.

#### Burden

Regarding the criteria of respondent burden, 81% (25/31) of patients reported spending 1 to 2 min while completing the ePROMs, whereas the mean time for in-clinic PROMs was 6 min (SD 2 min; range 3-9). All patients were able to learn the basic digital competencies needed to complete the ePROMs. No missing values were found using the two methods.

Regarding administrative burden, the total mean time spent per day by the pharmacist on mHeart was 33 min (SD 6 min; range 21-44). This time allowed follow-up of all the patients. The on-site PROMs required an office to be available and an average of 45 min for each individual assessment. Both methods required the professional to be trained in motivational interviewing, medication management, and transplant basics.

### Patient Satisfaction and Usability Survey

The completion rate was 93% (29/31 patients). The reasons for *no response* to the survey are detailed in Figure 1. HTxR reported no inconvenience because of the mHeart intervention approach employed. The mean ePROM appropriateness score was 8 (SD 2; score range: 0-10). The mean score for overall satisfaction with the mHeart approach was 9 (SD 2; score range: 0-10). All 100% (29/29) of the patients would recommend the mHeart platform to other recipients. Regarding patient suggestions for improving the platform, 24% (7/29) of HTxR made eight suggestions and 76% (22/29) of them responded, “No, I like it just as it is.”

Improvements were implemented based on patient feedback, for example, (1) to avoid patient recall bias, the order of the ePROM items was designed to automatically change whenever the test is completed; (2) patients could graphically consult any values they recorded in mHeart (eg, blood pressure); (3) pop-up alerts were established to let patients know that a new text message from the provider had arrived; and (4) diverse actions were implemented to decrease telephone use by patients to inquire about the compatibility of new therapies: the usability of the mHeart function to inquire about new therapies was improved, and text messages were sent to the patients, explaining how to use this function.

The details of each survey item score, patient suggestions, and the subsequent improvements are provided in Multimedia Appendix 11.

## Discussion

### Principal Findings

In this study, the first challenge was to validate mHeart ePROMs to detect early stage HTxR at risk for medication nonadherence in the home setting. With this aim in mind, the COSMIN [30] and SAC-MOS [31] standards provided a solid framework to support the quality of the assessed ePROM psychometric variables. As the mHeart ePROMs meet the minimum standards set by the ISOQOL [11], they can be used in clinical practice and comparative effectiveness research.

The excellent agreement observed between ePROMs and on-site PROMs confirmed that the mHeart electronic approach was as effective as the traditional on-site method in identifying medication nonadherence. The ePROMs used in mHeart showed multiple advantages over on-site PROMS, such as eliminating potential professional interpretation of ambiguous responses that could affect medication adherence rates. Furthermore, the electronic approach required fewer in-clinic facilities than the traditional method of assessing medication nonadherence. The integration of the mHeart data with the HIS also reduced the time required to record ePROM responses in patients’ medical records. These advantages reduced burden and enabled the pharmacists to focus on clinical tasks. This is clinically significant, as a pharmacist intervention is associated with better use of evidence-based therapies, reducing medication errors and emergency department visits while increasing patient satisfaction [58].

Regarding the baseline medication nonadherence values, the percentage of HTxR nonadherent to overall medication in this study was worrisome according to the Morisky-Green-Levine PROMs (32%) but similar to that observed in another series (33%) [59]. According to the SMAQ, the percentage (39%) of medication nonadherence to immunosuppressive treatment was slightly higher than the overall percentage reported in the HTx population (34%) [7] and considerably higher than that reported in a meta-analysis of the solid organ transplant population (25%) [4,25]. These results suggest that the ePROMs used in our study have a synergistic effect in identifying nonadherent recipients. However, they also highlight the need for intervention programs to improve medication nonadherence, as almost half of the HTxR were unaware of the consequences of medication nonadherence. Nevertheless, comparisons among medication nonadherence rates in the field of transplantation should be interpreted with caution as studies use different populations and methodologies [4].

The exploratory intervention program established to deal with this problem showed promising results. Immunosuppressive treatment adherence rates significantly improved in one-third of the recipients, according to the SMAQ. This figure is higher than that reported by most studies showing low or medium effect sizes of around 10% to 20% in medication adherence improvement [10,45]. In contrast to these studies, our program was designed to deliver personalized, internet-based multilevel interventions based on behavioral theories [7,21,22]. Indeed, human support and tailored interventions have been shown to be a requisite to improve medication nonadherence rates throughout eHealth [10,18]. Moreover, our exploratory study meets 72% of the TCS criteria (ie, items 1-11), indicating that the interventional study design complies with the theoretical basis of the intervention [32]. This is important as interventions meeting a minimum of 60% of the TCS criteria have been found to be highly effective [60].

Nonelectronic theory-based interventions have been considered highly effective when improvement was >20% [45]. Therefore, as the improvement in medication nonadherence in our exploratory study was higher (30%), the strategies applied proved to be synergistic and to enhance the effectiveness of the program [21,32]. Equally important, patients adhered well to the study protocol and provided excellent feedback. Among the benefits of the mHeart approach, patients highlighted personalized communication, support from professionals, and self-empowerment, which were the most relevant criteria used to design the mHeart intervention program.

### Limitations

Our study includes a limited but representative sample comprising 86% of all early stage HTxR in our center. This characteristic is common in transplant population, as the prevalence is limited [61]. We did not enroll chronic-stage recipients for the following reasons: (1) early posttransplant medication nonadherence is a high-risk behavior with a huge impact on survival [1], (2) we wanted to avoid wide heterogeneity in chronic-stage providers and treatments, and (3) we wanted to avoid chronic-stage recipients having to travel to the clinic for the study. In addition, although early stage recipients are typically better adherers [3,62], this did not prevent us from observing an effect in the highest risk period after transplant.

The interval between medication nonadherence assessments may have led to recall bias. Although this bias could have influenced the electronic score, this limitation is intrinsically related to the validation methodology to ensure that the electronic and traditional methods are performed in similar conditions and in patients with similar psychological and functional status. Moreover, the short study periods used were methodologically grounded according to the main study aim of validating the ePROMs. In *sensitivity to change measures*, a 1-month interval is considered an adequate interval to measure the validity of an indirect smartphone health measure [51,52]. Moreover, fortnightly assessments are sufficient to identify additional medication nonadherence in the transplant population [46]. In *reproducibility measures*, intervals of 1 to 2 weeks are common [63]. Therefore, a 7-day interval was selected to minimize the effect of possible confounding variables [31] related to the multifaceted factors affecting posttransplant medication nonadherence [10,46].

### Long-Term Workflow and Clinical Applications

Adherence monitoring is recognized as a standard quality practice in transplant centers [7]. In the past few decades, there has been a growing interest in improving the screening opportunities of medication nonadherence without increasing the in-clinic burden. The quality of the ePROM psychometric variables and the patient satisfaction reported in this study support the scalability of the mHeart ePROM for use in clinical practice and research [11]. The results obtained indicate that the electronic self-reporting approach provides a highly sensitive medication nonadherence measure in the transplant population to complement traditional and more time-consuming methods, such as blood tests or medication refills [25,46].

Furthermore, given that medication nonadherence behavior in the transplant population is influenced by several factors [7], optimal daily adherence is a real challenge for recipients [64]. Consequently, feasible holistic strategies are needed to help recipients reduce the negative impact of medication nonadherence on health outcomes [20]. The exploratory results of this behavioral theory–based intervention on medication nonadherence rates are encouraging. Future studies will determine the intervention’s effectiveness on clinical outcomes [13]. With this aim in mind, the EMERGE [28], TCS [32], and CONSORT-EHEALTH reporting criteria [17] standards were followed to support the scalability of the intervention methodology used in larger research. For now, the feasibility and effectiveness found in this study encourage following this path to curb the widespread problem of medication nonadherence.

### Conclusions

The electronic method implemented in the mHeart medical device successfully identified medication nonadherence in the HTx population. ePROMs demonstrated their potential to overcome the limitations of traditional on-site methods. The ePROMs’ quality properties supported their widespread use in research and clinical practice. The theory-based intervention program showed an encouraging improvement in medication adherence rates, with excellent patient satisfaction and usability scores. Therefore, the mHeart program resulted in significant benefits in estimating medication nonadherence in the HTx population and showed promise in guiding professionals’ interventions with the potential to optimize HTx outcomes.

## Appendix

Multimedia Appendix 1 Questionnaire designed for completion in face-to-face interviews: sociodemographic, clinical, and technology acceptance among the heart transplant recipients included in the Val-mHeart study.

Multimedia Appendix 2 Questionnaire designed to be administered in face-to-face interviews: patient-reported outcomes related to the treatment regimen.

Multimedia Appendix 3 Behavior change techniques selected for use during the behavior-based intervention program and workflow adapted for delivery using the mHeart platform, and a description of the mHeart-based treatment designed to improve medication adherence in the Val-mHeart study.

Multimedia Appendix 4 Sample video of the mHeart mobile app.

Multimedia Appendix 5 Electronic version of the Haynes-Sackett questionnaire, including 6 additional responses by patients to improve provider understanding of their difficulties with medication adapted for use with the mHeart platform.

Multimedia Appendix 6 Patient Satisfaction and Usability Survey Original Spanish Version (the results and the items' translation to English language are provided in Multimedia Appendix 11).

Multimedia Appendix 7 Methodology used to measure validity properties of the electronic patient reported outcome measures to assess medication adherence in the at-home setting using the mHeart platform in heart transplant recipients (The Val-mHeart Study).

Multimedia Appendix 8 Demographic and clinical characteristics of the early-stage heart transplant recipients included in the Val-mHeart Study.

Multimedia Appendix 9 Heart transplant recipients’ therapeutic characteristics and treatment-related patient-reported outcomes.

Multimedia Appendix 10 Nonadherence rates in early-stage heart transplant recipients listed by item in the study period.

Multimedia Appendix 11 Results of the Web-based survey on usability and satisfaction with the mHeart platform and intervention implemented during the study period in heart transplant recipients. Table 11A shows the categorical variables and Table 11B shows the quantitative variables.

## Abbreviations

CONSORT-EHEALTH: Consolidated Standards of Reporting Trials of Electronic and Mobile Health Applications and OnLine TeleHealth
COSMIN: COnsensus-based Standards for the selection of health Measurement Instruments
EMERGE: European Society for Patient Adherence, COMpliance, and Persistence Medication Adherence Reporting Guideline
ePROM: electronic patient-reported outcome measure
HIS: hospital information system
HSCSP: Hospital de la Santa Creu i Sant Pau, Barcelona, Spain
HTx: heart transplant
HTxR: heart transplant recipients
ISOQOL: International Society for Quality of Life Research
ISRII: International Society for Research on Internet Interventions
MNA: medication nonadherence
SAC-MOS: Scientific Advisory Committee of the Medical Outcomes Trust
SMAQ: Simplified Medication Adherence Questionnaire
TCS: Theory Coding Scheme

## References

[1] Dobbels F, de Geest S, van Cleemput J, Droogne W, Vanhaecke J (2004). Effect of late medication non-compliance on outcome after heart transplantation: a 5-year follow-up. J Heart Lung Transplant.

[2] Brocks Y, Zittermann A, Grisse D, Schmid-Ott G, Stock-Gießendanner S, Schulz U, Brakhage J, Benkler A, Gummert J, Tigges-Limmer K (2017). Adherence of heart transplant recipients to prescribed medication and recommended lifestyle habits. Prog Transplant.

[3] Korb-Savoldelli V, Sabatier B, Gillaizeau F, Guillemain R, Prognon P, Bégué D, Durieux P (2010). Non-adherence with drug treatment after heart or lung transplantation in adults: a systematic review. Patient Educ Couns.

[4] Dew MA, DiMartini AF, Dabbs AV, Myaskovsky L, Steel J, Unruh M, Switzer GE, Zomak R, Kormos RL, Greenhouse JB (2007). Rates and risk factors for nonadherence to the medical regimen after adult solid organ transplantation. Transplantation.

[5] Hansen R, Seifeldin R, Noe L (2007). Medication adherence in chronic disease: issues in posttransplant immunosuppression. Transplant Proc.

[6] Hugon A, Roustit M, Lehmann A, Saint-Raymond C, Borrel E, Hilleret M, Malvezzi P, Bedouch P, Pansu P, Allenet B (2014). Influence of intention to adhere, beliefs and satisfaction about medicines on adherence in solid organ transplant recipients. Transplantation.

[7] Denhaerynck K, Berben L, Dobbels F, Russell CL, Crespo-Leiro MG, Poncelet AJ, de Geest S, BRIGHT study team (2018). Multilevel factors are associated with immunosuppressant nonadherence in heart transplant recipients: the international BRIGHT study. Am J Transplant.

[8] Gomis-Pastor M, Mingell ER, Perez SM, Loidi VB, Lopez LL, Bassons AD, Pousa AA, Ribera AF, Ferrero-Gregori A, Perich LG, Bafalluy MA (2019). Multimorbidity and medication complexity: new challenges in heart transplantation. Clin Transplant.

[9] Senft Y, Kirsch M, Denhaerynck K, Dobbels F, Helmy R, Russell CL, Berben L, de Geest S, BRIGHT study team (2017). Practice patterns to improve pre and post-transplant medication adherence in heart transplant centres: a secondary data analysis of the international BRIGHT study. Eur J Cardiovasc Nurs.

[10] Kini V, Ho PM (2018). Interventions to improve medication adherence: a review. J Am Med Assoc.

[11] Reeve BB, Wyrwich KW, Wu AW, Velikova G, Terwee CB, Snyder CF, Schwartz C, Revicki DA, Moinpour CM, McLeod LD, Lyons JC, Lenderking WR, Hinds PS, Hays RD, Greenhalgh J, Gershon R, Feeny D, Fayers PM, Cella D, Brundage M, Ahmed S, Aaronson NK, Butt Z (2013). ISOQOL recommends minimum standards for patient-reported outcome measures used in patient-centered outcomes and comparative effectiveness research. Qual Life Res.

[12] Sarzynski E, Decker B, Thul A, Weismantel D, Melaragni R, Cholakis E, Tewari M, Beckholt K, Zaroukian M, Kennedy AC, Given C (2017). Beta testing a novel smartphone application to improve medication adherence. Telemed J E Health.

[13] Ritterband LM, Andersson G, Christensen HM, Carlbring P, Cuijpers P (2006). Directions for the International Society for Research on Internet Interventions (ISRII). J Med Internet Res.

[14] Griffiths F, Lindenmeyer A, Powell J, Lowe P, Thorogood M (2006). Why are health care interventions delivered over the internet? A systematic review of the published literature. J Med Internet Res.

[15] Heron KE, Smyth JM (2010). Ecological momentary interventions: incorporating mobile technology into psychosocial and health behaviour treatments. Br J Health Psychol.

[16] Free C, Phillips G, Galli L, Watson L, Felix L, Edwards P, Patel V, Haines A (2013). The effectiveness of mobile-health technology-based health behaviour change or disease management interventions for health care consumers: a systematic review. PLoS Med.

[17] Eysenbach G, CONSORT-EHEALTH Group (2011). CONSORT-EHEALTH: improving and standardizing evaluation reports of Web-based and mobile health interventions. J Med Internet Res.

[18] Lancaster K, Abuzour A, Khaira M, Mathers A, Chan A, Bui V, Lok A, Thabane L, Dolovich L (2018). The use and effects of electronic health tools for patient self-monitoring and reporting of outcomes following medication use: systematic review. J Med Internet Res.

[19] Mira JJ, Navarro I, Botella F, Borrás F, Nuño-Solinís R, Orozco D, Iglesias-Alonso F, Pérez-Pérez P, Lorenzo S, Toro N (2014). A Spanish pillbox app for elderly patients taking multiple medications: randomized controlled trial. J Med Internet Res.

[20] Fleming JN, Taber DJ, McElligott J, McGillicuddy JW, Treiber F (2017). Mobile health in solid organ transplant: the time is now. Am J Transplant.

[21] Davis R, Campbell R, Hildon Z, Hobbs L, Michie S (2015). Theories of behaviour and behaviour change across the social and behavioural sciences: a scoping review. Health Psychol Rev.

[22] Conn VS, Enriquez M, Ruppar TM, Chan KC (2016). Meta-analyses of theory use in medication adherence intervention research. Am J Health Behav.

[23] Salvo MC, Cannon-Breland ML (2015). Motivational interviewing for medication adherence. J Am Pharm Assoc (2003).

[24] Miller WR, Rose GS (2009). Toward a theory of motivational interviewing. Am Psychol.

[25] Fine RN, Becker Y, de Geest S, Eisen H, Ettenger R, Evans R, Rudow DL, McKay D, Neu A, Nevins T, Reyes J, Wray J, Dobbels F (2009). Nonadherence consensus conference summary report. Am J Transplant.

[26] Vitinius F, Ziemke M, Albert W (2015). Adherence with immunosuppression in heart transplant recipients. Curr Opin Organ Transplant.

[27] Froud R, Fawkes C, Foss J, Underwood M, Carnes D (2018). Responsiveness, reliability, and minimally important and minimal detectable changes of 3 electronic patient-reported outcome measures for low back pain: validation study. J Med Internet Res.

[28] de Geest S, Zullig LL, Dunbar-Jacob J, Helmy R, Hughes DA, Wilson IB, Vrijens B (2018). ESPACOMP Medication Adherence Reporting Guideline (EMERGE). Ann Intern Med.

[29] Mokkink LB, Terwee CB, Patrick DL, Alonso J, Stratford PW, Knol DL, Bouter LM, de Vet HC (2010). The COSMIN checklist for assessing the methodological quality of studies on measurement properties of health status measurement instruments: an international Delphi study. Qual Life Res.

[30] Mokkink LB, Terwee CB, Patrick DL, Alonso J, Stratford PW, Knol DL, Bouter LM, de Vet HC (2010). The COSMIN study reached international consensus on taxonomy, terminology, and definitions of measurement properties for health-related patient-reported outcomes. J Clin Epidemiol.

[31] Aaronson N, Alonso J, Burnam A, Lohr KN, Patrick DL, Perrin E, Stein RE (2002). Assessing health status and quality-of-life instruments: attributes and review criteria. Qual Life Res.

[32] Michie S, Prestwich A (2010). Are interventions theory-based? Development of a theory coding scheme. Health Psychol.

[33] Eysenbach G (2004). Improving the quality of web surveys: the Checklist for Reporting Results of Internet E-Surveys (CHERRIES). J Med Internet Res.

[34] Vrijens B, de Geest S, Hughes DA, Przemyslaw K, Demonceau J, Ruppar T, Dobbels F, Fargher E, Morrison V, Lewek P, Matyjaszczyk M, Mshelia C, Clyne W, Aronson JK, Urquhart J, Team AB (2012). A new taxonomy for describing and defining adherence to medications. Br J Clin Pharmacol.

[35] Abraham C, Michie S (2008). A taxonomy of behavior change techniques used in interventions. Health Psychol.

[36] Baumel A, Kane JM (2018). Examining predictors of real-world user engagement with self-guided eHealth interventions: analysis of mobile apps and websites using a novel dataset. J Med Internet Res.

[37] Muench F, Baumel A (2017). More than a text message: dismantling digital triggers to curate behavior change in patient-centered health interventions. J Med Internet Res.

[38] Trilema Salud.

[39] Socioemprende SL Google Play.

[40] Socioemprende App Store - Apple.

[41] Gomis-Pastor M, Mangues M, Pellicer V, Ors M (2019). Mendeley Data.

[42] Sackett DL, Snow JC, Haynes RB, Taylor DW, Sackett DL (1979). The magnitude of compliance and non-compliance. Compliance in Health Care.

[43] Sackett DL, Haynes RB, Guyat GH, Haynes RB, Sackett DL, Guyatt GH, Tugwell P (1994). Ayudar a los pacientes a cumplir los tratamientos. Epidemiol clínica, Cienc básica para la Med clínica 2a ed.

[44] Morisky DE, Green LW, Levine DM (1986). Concurrent and predictive validity of a self-reported measure of medication adherence. Med Care.

[45] Dobbels F, de Bleser L, Berben L, Kristanto P, Dupont L, Nevens F, Vanhaecke J, Verleden G, de Geest S (2017). Efficacy of a medication adherence enhancing intervention in transplantation: The MAESTRO-Tx trial. J Heart Lung Transplant.

[46] Gustavsen MT, Midtvedt K, Lønning K, Jacobsen T, Reisaeter AV, de Geest S, Andersen MH, Hartmann A, Åsberg A (2019). Evaluation of tools for annual capture of adherence to immunosuppressive medications after renal transplantation - a single-centre open prospective trial. Transpl Int.

[47] Val Jiménez A, Amorós Ballestero G, Martínez PF (1992). Estudio descriptivo del cumplimiento del tratamiento farmacológico antihipertensivo y validación del test de Morisky y Green. Estudio descriptivo del cumplimiento del tratamiento farmacológico antihipertensivo y validación del test de Morisky y Green.

[48] Orozco-Beltrán D, Carratalá-Munuera C, Gil-Guillén V (2015). Improving treatment adherence: one of the most effective ways of increasing patient survival in secondary prevention. Rev Esp Cardiol Supl.

[49] Conthe P, Tejerina F (2007). Heart failure treatment adherence and quality of life. Rev Esp Cardiol Supl.

[50] Suárez FJ, Plumed JS, Valentín MA, Palomo PP, Cepeda MA, Aguiar DL, Grupo de Estudio Vatren (2011). Validation on the simplified medication adherence questionnaire (SMAQ) in renal transplant patients on tacrolimus. Nefrologia.

[51] Conthe P, Contreras EM, Pérez AA, García BB, Martín MN, Jurado MG, Baturone MO, Pinto JL (2014). Treatment compliance in chronic illness: current situation and future perspectives. Rev Clin Esp.

[52] Pan YC, Lin HH, Chiu YC, Lin SH, Lin YH (2019). Temporal stability of smartphone use data: determining fundamental time unit and independent cycle. JMIR Mhealth Uhealth.

[53] Viladrich C, Doval E (2011). Medición: Fiabilidad y Validez.

[54] Akoglu H (2018). User's guide to correlation coefficients. Turk J Emerg Med.

[55] Fleiss JL, Levin B, Myunghee CP (1981). Statistical Methods for Rates & Proportions. Third Edition.

[56] Hogan TP, Benjamin AM, Brezinski KL (2000). Reliability methods: a note on the frequency of use of various types. Educ Psychol Meas.

[57] Gorsuch RL (1983). Factor Analysis. Second Edition.

[58] Milfred-LaForest SK, Chow SL, DiDomenico RJ, Dracup K, Ensor CR, Gattis-Stough W, Heywood JT, Lindenfeld J, Page RL, Patterson JH, Vardeny O, Massie BM (2013). Clinical pharmacy services in heart failure: an opinion paper from the Heart Failure Society of America and American College of Clinical Pharmacy Cardiology Practice and Research Network. Pharmacotherapy.

[59] Pérez AB, Suárez AL, Rodríguez JR, Márquez JM, Gallé EL (2013). Medication adherence in patients who undergo cardiac transplantation. Transplant Proc.

[60] Lycett HJ, Raebel EM, Wildman EK, Guitart J, Kenny T, Sherlock J, Cooper V (2018). Theory-based digital interventions to improve asthma self-management outcomes: systematic review. J Med Internet Res.

[61] de Bleser L, Matteson M, Dobbels F, Russell C, de Geest S (2009). Interventions to improve medication-adherence after transplantation: a systematic review. Transpl Int.

[62] de Geest S, Burkhalter H, Bogert L, Berben L, Glass TR, Denhaerynck K, Psychosocial Interest Group, Swiss Transplant Cohort Study (2014). Describing the evolution of medication nonadherence from pretransplant until 3 years post-transplant and determining pretransplant medication nonadherence as risk factor for post-transplant nonadherence to immunosuppressives: the Swiss Transplant Cohort Study. Transpl Int.

[63] Polit DF (2014). Getting serious about test-retest reliability: a critique of retest research and some recommendations. Qual Life Res.

[64] Vanhoof JM, Vandenberghe B, Geerts D, Philippaerts P, de Mazière P, Dabbs AD, de Geest S, Dobbels F, PICASSO-Tx consortium (2018). Shedding light on an unknown reality in solid organ transplant patients' self-management: a contextual inquiry study. Clin Transplant.

