# Supplementary material for: A Mobile App (mHeart) to Detect Medication Nonadherence in the Heart Transplant Population: Validation Study
Source: JMIR Mhealth Uhealth. 2020 Feb 4;8(2):e15957. doi: 10.2196/15957 (PMC7055830; doi:10.2196/15957)
Supplement: Multimedia Appendix 1 [file mhealth_v8i2e15957_app1.pdf]

This is a Multimedia Appendix to a full manuscript published in the J Med Internet Res.
For full copyright and citation information see http://dx.doi.org/10.2196/jmir.xxxx

Multimedia Appendix 1. Questionnaire designed for completion in face-to-face interviews: sociodemographic, clinical and technology acceptance among the heart transplant recipients included in the Val-mHeart study

Has the patient signed the informed consent form? Yes	Interview date by MG: __/__/____
Does the patient meet the inclusion criteria[a]? Yes	Prospective review of the patient EHR: __/__/____
Sociodemographic and clinical variables	
Patient's HSCSP code: __________<br>Date of birth: __/__/____<br>Gender<br>☐ Male<br>☐ Female<br>☐ Other<br>Weight: ___ kilograms<br>Height: ___ meters<br>Date of heart transplant: __/__/___<br>Urgent HTx: Yes / No	Heart failure etiology:<br>☐ Congenital<br>☐ Coronary/ischemic<br>☐ Myocarditis<br>☐ Cardiomyopathy<br>☐ Valvular cardiac disease<br>☐ Hypertrophic cardiomyopathy<br>☐ Re-transplant<br>☐ Other
Educational attainment:<br>☐ No formal education<br>☐ Middle school graduate<br>☐ High school graduate<br>☐ University graduate	Employment status:<br>☐ Temporary medical leave<br>☐ Long-term disability<br>☐ Retired<br>☐ No previous employment<br>☐ Currently employed
Need or requirement for caregiver: Yes / No	Lives with someone else: Yes / No
Number of comorbidities (EHR and confirmed by the patient): ____	
Type of comorbidity post-transplant (more than 1 answer allowed):<br>☐ High blood pressure<br>☐ Dyslipidemia<br>☐ Chronic kidney failure<br>☐ Osteopathies and chondroplasties<br>☐ Diseases of the nervous system<br>☐ Mood and anxiety disorders	☐ Digestive system diseases or disorders<br>☐ Diabetes mellitus<br>☐ Neoplasia<br>☐ Arthropathies<br>☐ Others: ______________________________<br>______________________________
Total number of drugs prescribed (EHR and confirmed by the patient): ____<br>Over-the-counter drugs (not included in the medical prescription): ____	
Technology acceptance and technical support variables	
Do you think it would be useful for you to use an app or website designed by your transplant team to improve your home-based management and therapy follow-up?	☐ Not very useful<br>☐ Useful<br>☐ Very useful<br>☐ Not yet known until the platform is tested
How frequently do you use technology?	☐ Often<br>☐ Occasionally<br>☐ Never
Do you use technology for health purposes?	☐ Yes<br>☐ No<br>☐ Don't know/No answer
Do you think you may require assistance in using the platform?	☐ Yes<br>☐ No<br>☐ Not yet known until the platform is tested
How much time did you spend in answering the mHeart medication adherence test? (Only applies in Visit 2)	☐ 1-2 minutes<br>☐ 3-5 minutes<br>☐ >5 minutes<br>☐ Don't know/No answer

EHR, Electronic Health Records; Tx, transplant.
[a] Inclusion criteria: early-stage (<1.5 years post-HTx) adult patients without severe decompensation or cognitive impairment.
