# Supplementary material for: A Mobile App (mHeart) to Detect Medication Nonadherence in the Heart Transplant Population: Validation Study
Source: JMIR Mhealth Uhealth. 2020 Feb 4;8(2):e15957. doi: 10.2196/15957 (PMC7055830; doi:10.2196/15957)
Supplement: Multimedia Appendix 2 [file mhealth_v8i2e15957_app2.pdf]

This is a Multimedia Appendix to a full manuscript published in the J Med Internet Res.
For full copyright and citation information see http://dx.doi.org/10.2196/jmir.xxxx

Multimedia Appendix 2. Questionnaire designed to be administered in face-to-face interviews: patient-reported outcomes (PROs) related to the treatment regimen

Has the patient signed the informed consent form? Yes	Interview date by MG: __/__/___
Does the patient meet the inclusion criteria[a]? Yes	Prospective review of the patient EHR: __/__/___

Medication adherence in-clinic instruments

Haynes-Sackett test[a]

Most patients have difficulty taking all their tablets, do you have difficulties taking all of yours? Yes / No

Medication Adherence Validated Test: Morisky-Green-Levine Scale[1][a]

**1.**	Do you ever forget to take your medication?	☐ Yes	☐ No
**2.**	Are you careless at times about taking your medication?	☐ Yes	☐ No
**3.**	When you feel better, do you sometimes stop taking your medication?	☐ Yes	☐ No
**4.**	Sometimes, if you feel worse when you take your medication, do you stop taking it?	☐ Yes	☐ No

Medication Adherence Validated Test: Simplified Medication Adherence Questionnaire (SMAQ) transplant patients[a]

This questionnaire refers to your level of adherence with the immunosuppressive treatment prescribed by your doctor after your transplant. Please answer all of the questions, indicating the correct response in each case. Please remember that your answers are confidential, and that you should respond to the questions in as truthful a manner as possible. Thank you.

1.	Do you always take your medication at the appropriate time?	☐ Yes	☐ No
2.	When you feel bad, have you ever discontinued taking your medication?	☐ Yes	☐ No
3.	Have you ever forgotten to take your medication?	☐ Yes	☐ No
4.	Have you ever forgotten to take your medication during the weekend?	☐ Yes	☐ No
5.	In the LAST WEEK, HOW MANY TIMES did you fail to take your prescribed dose?	☐ Never<br>☐ 1-2 times<br>☐ 3-5 times	☐ 6-10 times<br>☐ >10 times
6.	SINCE YOUR LAST VISIT, how many whole days have gone by in which you did not take your medication?	☐ Nº of days ___	

Patient's management, knowledge and beliefs about medication

The patient prepares and takes his/her medication autonomously: Yes/No

Reasons for lack of autonomy with medication (free field)

Knowledge of the therapeutic regimen

- Total number of drugs prescribed. State the drug and regimen: __
- Drugs remembered (names): __
- Doses remembered: __
- Intakes remembered: __
- Indications remembered: __

Degree of inconvenience perceived by the patient related to taking his/her medication as prescribed every day (scale 0-10)

Patient's perception of taking excessive medication: Yes / No

Patient's awareness of the importance of immunosuppressive therapy

1. If you discontinued taking your immunosuppressants completely, what do you think would happen to you?
   - ☐ Nothing
   - ☐ I don't know
   - ☐ A different answer involving rejection
2. If you sometimes forgot to take your immunosuppressants, what do you think would happen to you?
   - ☐ Nothing
   - ☐ I don't know
   - ☐ A different answer involving rejection
3. Did you modify the immunosuppressant timetable in the last week?
   - ☐ No
   - ☐ >1 day
   - ☐ I don't remember
4. Did you modify the immunosuppressant timetable since the last visit?
   - No
   - On >1 day
   - On >5 days
   - Don't remember

Type of side effect reported by patients

- ☐ Visual impairment
- ☐ Psychological: emotional disorders, insomnia
- ☐ Neurological: tremor, dizziness, headache
- ☐ Mucosa and aesthetic: thrush, gingival disorder, alopecia, increased hair growth, visual disorders
- ☐ Pain: muscular pain, joint pain
- ☐ Gastric: diarrhea, nausea, constipation, vomiting
- ☐ Muscular: weariness, tiredness or fatigue, cramps
- ☐ Other: ______________________________

EHR, electronic health records; Tx, transplant.

[a] Non-adherence to medications in the implementation phase is defined as "actual dosing does not correspond to the prescribed dosing regimen due to delays, omissions or extra doses" and is measured by self-report questionnaires. A patient is classified as non-adherent if he/she responds to any of the questions with an answer indicating non-adherence. Delays refer to irregularities with the intake schedule (±2 hours).
