# Supplementary material for: A Mobile App (mHeart) to Detect Medication Nonadherence in the Heart Transplant Population: Validation Study
Source: JMIR Mhealth Uhealth. 2020 Feb 4;8(2):e15957. doi: 10.2196/15957 (PMC7055830; doi:10.2196/15957)
Supplement: Multimedia Appendix 3 [file mhealth_v8i2e15957_app3.pdf]

This is a Multimedia Appendix to a full manuscript published in the J Med Internet Res.
For full copyright and citation information see http://dx.doi.org/10.2196/jmir.xxxx

Multimedia Appendix 3

3.1. Behavior change techniques selected for use during the theory-based intervention program and workflow adapted for delivery using the mHeart platform

Technique (theoretical framework a)	Description of the behavioral intervention technique and the *element* b to support the strategy	Timing c	Dose frequency d
Motivational communication skills (MI)	Use of any patient-professional communication opportunity to prompt the patient to provide self-motivating statements and self-evaluations: (1) minimize resistance to change; (2) maintain the change achieved. *Element: I,N, M, R, V, C.*	(1) & (2)<br>Adh & N-Adh	(1) & (2)<br>Continuously
Tailoring	Use any opportunity to individualize the management of adherence to a specific patient, based on the patient's environment and self-reports. *Element: all.*	Adh & N-Adh	Continuously
Provide instructions (SCogT)	Tell the patient how to adopt a medication-related behavior, i.e. (1) education on the importance of taking immunosuppressive medication and management of side effects. *Element: I, M, C*; (2) responses to the patient's queries and doubts. *Element: M*; (3) information about the prescription change (doses, drug, etc.) and explaining the reason for the change. *Element: M,C, N.*	(1), (2) & (3)<br>Adh & N-Adh	(1) Baseline & If Need<br>(2) & (3) Continuously
Time management	(1) Find a time for intake that fits with each patient's lifestyle. Include this schedule in the patient's mHeart agenda and activate intake alarms if necessary. (2) Train him/her on what to do if intake is late. *Element: A, M, I*	(1) & (2)<br>Adh & N-Adh	(1) & (2)<br>Baseline & If Need
Goal setting (CT)	Involve the professional and recipients in detailed planning of the steps the patient will take to acquire the medication skills needed for adequate medication adherence (frequency, intensity, duration and context). *Element: all.*	Adh & N-Adh	Baseline & If Need
Provide information on the behavior-health link (IMB)	Consider providing general information by mass campaigns about behavioral risk, i.e. (1) importance of taking immunosuppressive drugs on time, (2) reminding patients about sun protection adherence. *Element: M*	(1) & (2)<br>Adh & N-Adh	(1) Baseline & W2<br>(2) W2
Provide information on the consequences (CT)	Inform the patient of the benefits and costs of changing or not changing a behavior (i.e. adherence or non-adherence to medications or monitoring). E.g. pressure rates on range if adhere to antihypertensives. *Elements: M, C, I, R, V*	Adh & N-Adh	Once/week & If needed
Prompt self-monitoring of behavior (CT)	Prompt patient to report data related to medication behavior, i.e. (1) drug intake in the agenda; (2) medication adherence ePROMs[e]; (3) side effects; (4) glycemia, blood pressure, etc. *Elements: L, M, V, R, P, N*	(2), (3) & (4)<br>Adh & N-Adh<br>(1) N-Adh	(1) (2) (4)<br>Continuously<br>(3) If need
Provide feedback on performance (CT)	Provide the patient with data based on the self-reported information to maintain patient motivation and adherence with the intervention program, i.e. (1) biomesure pattern; (2) side effects; (3) medication adherence ePROMs[e]; (4) medication intake. *Elements: P, R, V, M*	(1), (2), (3) & (4)<br>Adh & N-Adh	(1) & (4)<br>Once/week<br>(2) If needed<br>(3) Continuously
Provide contingent rewards (OC)	Provide praise or encouragement linked to the achievement of specified behaviors, e.g. praise any improvement in self-management. *Elements: P, R, V, M*	Adh & N-Adh	Continuously
Prompt review of behavioral goals (CT)	Review the intentions or goals previously agreed with the patient; discuss and readjust the plan if necessary. *Elements: M, C*	N-Adh	Once/week & If need
Identify barriers to behavior (SCogT)	Identify the barriers to adequate adherence using PROMs, e.g. detect a specific side effect reported by the patient electronically. *Elements: R*	N-Adh	If needed
Action planning & problem solving	Plan ways of overcoming the barriers detected and reach an agreement with the patient, e.g. discuss medication beliefs with the patient. *Elements: all*	N-Adh	If needed
Environmental restructuring	Provide guidance to change the patient's habits that could hamper medication adherence. *Elements: all*	N-Adh	If needed
Teaching the use of prompts/cues (OC)	Teach the patient to identify environmental cues to remind him/her to adopt a behavior, i.e. (1) times of meals could serve as reminders of medication intake; (2) a beeping signal at the time of scheduled medication intake. *Element: A, P, M*	N-Adh	Baseline & If needed
Prompt intention formation (TRA,TPB, SCogT, IMB)	Encourage the patient to decide to act or set a general goal, e.g. to make a behavioral resolution such as "I will take my pills on time every day". *Element: I, N, L, M, V*	N-Adh	If needed
Prompting focus on past success	Discuss or review with the patient past behaviors related to negative outcomes. *Element: M, C, V, R*	N-Adh	If needed
Others' approval (TRA, TPB, IMB)	Provide information on what others think of a behavior, i.e. inform the patient that professionals will disapprove of an unhealthy behavior. *Element: M*	Continuously<br>N-Adh	
Provide information on others' behavior	Compare anonymous experiences, e.g., compare the patient's prescription with another significantly more complex regimen to reduce his/her feeling of burden. *Element: M, V*	N-Adh	If needed
Use follow-up prompts	Communicate the patient if a part of the intervention is complete, i.e. (1) several goals were planned; (2) a behavior has changed gradually. *Element: all*	(1) & (2)<br>N-Adh	If needed
Prompt identification as a role model	Indicate how the patient may be an example to others and influence their behavior, e.g., offer a patient to be part of the voluntary service. *Element: M*	Adh	W3

[a] The theoretical frameworks are: CT, control theory; IMB, information-motivation-behavioral skills model; MI, Motivational Interview; OC, operant conditioning; SCogT, social-cognitive theory; TPB, theory of planned behavior; TRA, theory of reasoned action.
[b] Elements (i.e. components or objects of the technology intended to implement the strategy) used in the study: (A) alerts, (P) prompts/reminders, (N) notifications, (M) messages, (L) logs, (R) reports, (V) visualizations, (C) video-calls, (I) Information delivery. [1,2]

[c] Non-adherence to medication in the implementation phase is defined as "actual dosing does not correspond to the prescribed dosing regimen due to delays, omissions or extra doses" and is measured by self-report questionnaires. Delays refer to irregularities with the intake schedule (±2 hours). Adh: medication-adherent recipient; N-adh: only if the patient is classified as non-adherence to medications (implementation phase).
[d] Baseline: when the treatment begins; Continuously: every time the task is scheduled during the treatment period between assessment 1 and 2; If Need: when provider detect that the strategy is needed based on reports or goals established; Once/week: at least 1 time per week based on reports; W1: during week 1 of the study; W2: during week 2 of the study; W3: during week 3 of the study.
[e] ePROMs, electronic patient-reported measures.

3.2. Description of mHeart-based treatment designed to improve medication adherence in the Val-mHeart study

The information complements the data in the Val-mHeart manuscript. For more information, please refer to the manuscript.

Specific aim of the treatment

- The treatment is defined as the interventional program applied in this study based on multiple internet-based strategies or interventions to achieve the clinical aim.
- The clinical aim of the treatment in the Val-mHeart study was to optimize medication adherence management in early-stage heart transplant recipients, i.e. to reduce the rate of non-adherent recipients.

Treatment duration

- A period of at least 1 month (between assessments 1 and 2).

Type of treatment

- The e-interventions were interactive with additional human support through the mHeart platform.
- The provider was a female clinical pharmacist with experience in motivational interviewing and specialized in heart transplants. The patients' first interaction with this provider was during hospitalization for the transplant procedure. No other contact was provided on-site after the first baseline study visit.
- The patients' characteristics are described in the manuscript, including the training and technical assistance received.

Delivery platform

- The hardware platform delivers the intervention via mobile platforms such as smartphones. Patients had access to a complementary website via desktop computers. Providers manage the platform through the website.
- Participants used their own cell phone and paid for their internet use. No incentives were provided for participation.
- The mHeart software (mobile application and website) is a Behavior Intervention Technology to facilitate the following overall goals: (1) health behavior change (i.e. increase patients' healthy behaviors and prevent the onset of disease); and (2) targeted disease management (i.e. facilitate therapeutic interventions and improve patients' self-management).
- The features specifically designed to manage medication adherence are provided in the manuscript. Other components or functionalities are detailed in a video of the mobile application provided in [Dataset][3].
- More information on developers, technical specifications and Source Code are provided in [Dataset][3].

Presentation strategy

- The mHeart platform is based on visual aids and minimizing text and passive information. For readers to have a clear sense of the aesthetics, visual aids used and other features, they were provided with a video with a demo trial of the clinical use of the app. Thus, readers can examine samples or portions of eHealth interventions through mHeart.
- Interactive **elements** were also used as digital **triggers** to prevent the law of attrition; i.e. (A) alerts, (P) prompts and reminders, (N) notifications, (M) messages, (L) logs, (R) reports, (V) visualizations, (C) video-calls, (I) information delivery. [1,2]

Content

- The interventional treatment **design** was based on published literature on internet-based interventions with impact on health behavior change, but also strategies to prevent patient attrition. [4–11]
- All the **behavioral change techniques** [12,13] used in the treatment are described based on Michie's taxonomy [14] and are provided in the Table. The most **important strategies** applied were human support, motivational engagement, therapeutic alliance strategies, [1,15] and individually-tailored feedback. [12,13,16–18] **Descriptions** of the strategies and examples are provided in the Table.
- The strategy could be aimed at (1) forming a behavior; (2) altering a behavior (3) reinforcing a behavior.
- The interventions were **tailored** based on mHeart patient-reported data collected using (1) dynamic information from the mHeart features, and (2) information collected in the in-clinic baseline interviews.
- Interventions were **delivered** using motivational interviewing skills. [19,20]

Workflows

- **Intended doses** and **optimal timing for the use** of each technique are also described in the Table.
- **Conditions of use**, a mixture of time-based, event-based or task-completion rules were applied as required. [21] Thus, the **complexity** of the strategies varied depending on the user and the task. A combination of these techniques was common.
- Video-calls were not scheduled and were limited to very occasional situations when a text message was insufficient to deliver highly complex information.

This is a Multimedia Appendix to a full manuscript published in the J Med Internet Res.
For full copyright and citation information see http://dx.doi.org/10.2196/jmir.xxxx

References

1. Baumel A, Kane JM. Examining Predictors of Real-World User Engagement with Self-Guided eHealth Interventions: Analysis of Mobile Apps and Websites Using a Novel Dataset. *J Med Internet Res*. 2018;20(12):e11491. PMID:30552077
2. Muench F, Baumel A. More Than a Text Message: Dismantling Digital Triggers to Curate Behavior Change in Patient-Centered Health Interventions. *J Med Internet Res*. 2017;19(5):e147. DOI:10.2196/jmir.7463
3. Gomis-Pastor M, Mangues MA, Pellicer V. mHeart - mHealthCare Platform Adapted to the Heart Transplant Population - Technical Specifications and Software Source Code. *Mendeley Data*. 2019;V2. DOI:10.17632/yf2dgcfmmh.2
4. Ritterband LM, Andersson G, Christensen HM, Carlbring P, Cuijpers P. Directions for the International Society for Research on Internet Interventions (ISRII). *J Med Internet Res*. 2006;8(3):1-6. PMID:17032639
5. Heron KE, Smyth JM. Ecological Momentary Interventions: Incorporating Mobile Technology Into Psychosocial and Health Behavior Treatments. *Br J Heal Psychol*. 2010;15(Pt 1):1-39. PMID:19646331
6. Free C, Phillips G, Galli L, Watson L, Felix L, Edwards P, et al. The Effectiveness of Mobile-Health Technology-Based Health Behaviour Change or Disease Management Interventions for Health Care Consumers: A Systematic Review. *PLoS Med*. 2013;10(1):e1001362. PMID:23349621
7. Whittaker R, Merry S, Dorey E, Maddison R. A development and evaluation process for mhealth interventions: Examples from New Zealand. *J Health Commun*. 2012;17:11-21. PMID:22548594
8. Yen P-Y, Bakken S. Review of health information technology usability study methodologies. *J Am Med Inf Assoc*. 2012;19(3):413-422. PMID:21828224
9. Eysenbach G, CONSORT-EHEALTH Group. CONSORT-EHEALTH: improving and standardizing evaluation reports of Web-based and mobile health interventions. *J Med Internet Res*. 2011;13(4). PMID:22209829
10. Kohl LFM, Crutzen R, De Vries NK. Online prevention aimed at lifestyle behaviors: A systematic review of reviews. *J Med Internet Res*. 2013;15(7):1-13. DOI:10.2196/jmir.2665
11. Kelders SM, Kok RN, Ossebaard HC, Van Gemert-Pijnen JEWC. Persuasive system design does matter: A systematic review of adherence to web-based interventions. *J Med Internet Res*. 2012;14(6):1-24. DOI:10.2196/jmir.2104
12. Davis R, Campbell R, Hildon Z, Hobbs L, Michie S. Theories of behaviour and behaviour change across the social and behavioural sciences: a scoping review. *Health Psychol Rev*. 2015;9(3):323-344. PMID:25104107
13. Conn VS, Enriquez M, Ruppar TM, Chan KC. Meta-analyses of Theory Use in Medication Adherence Intervention Research. *Am J Health Behav*. 2016;40(2):155-171. PMID:26931748
14. Abraham C, Michie S. A Taxonomy of Behavior Change Techniques Used in Interventions. *Heal Psychol*. 2008;27(3):379-387. PMID:18624603
15. Pagès-Puigdemont N, Mangues MA, Masip Giovanna M, Fernández-Maldonado GL, Blancafort S, Tuneu L. Patients' Perspective of Medication Adherence in Chronic Conditions: A Qualitative Study. *Adv Ther*. 2016;33(10):1740-1754. PMID:27503082
16. Webb TL, Joseph J, Yardley L, Michie S. Using the Internet to promote health behavior change: A systematic review and meta-analysis of the impact of theoretical basis, use of behavior change techniques, and mode of delivery on efficacy. *J Med Internet Res*. 2010;12(1):1-18. DOI:10.2196/jmir.1376
17. Kok G, Schaalma H, Ruiter RAC, Van Empelen P, Brug J. Intervention Mapping: A Protocol for Applying Health Psychology Theory to Prevention Programmes. *J Health Psychol*. 2004;9(1):85-98. DOI:10.1177/1359105304038379
18. Michie S, Johnston M, Francis J, Hardeman W, Eccles M. From Theory to Intervention: Mapping Theoretically Derived Behavioural Determinants to Behaviour Change Techniques. *Appl Psychol*. 2008;57(4):660-680. DOI:10.1111/j.1464-0597.2008.00341.x
19. Salvo MC, Cannon-Breland ML. Motivational interviewing for medication adherence. *J Am Pharm Assoc*. 2015;55(4):e354-e363. PMID:26161493
20. Miller WR, Rose GS. Toward a Theory of Motivational Interviewing MI was not founded on theory. *Am Psychol*. 2009;64(6):527-537. PMID:19739882
21. Mohr DC, Schueller SM, Montague E, Burns MN, Rashidi P. The behavioral intervention technology model: An integrated conceptual and technological framework for ehealth and mhealth interventions. *J Med Internet Res*. 2014;16(6). PMID:24905070
