# Supplementary material for: A Mobile App (mHeart) to Detect Medication Nonadherence in the Heart Transplant Population: Validation Study
Source: JMIR Mhealth Uhealth. 2020 Feb 4;8(2):e15957. doi: 10.2196/15957 (PMC7055830; doi:10.2196/15957)
Supplement: Multimedia Appendix 5 [file mhealth_v8i2e15957_app5.pdf]

This is a Multimedia Appendix to a full manuscript published in the J Med Internet Res.
For full copyright and citation information see http://dx.doi.org/10.2196/jmir.xxxx

Multimedia Appendix 5. Electronic version of the Haynes-Sackett questionnaire including 6 additional responses by patients to improve provider understanding of their difficulties with medication adapted for use with the mHeart platform

**Item 1. Most patients have difficulty taking all their tablets. Do you have difficulties taking yours? Please select an option…**
☐ No, I do not have any difficulties
☐ Yes,
☐ Yes, I sometimes forget to take my medication
☐ Yes, I lack information about the medication and/or the disease
☐ Yes, I feel demotivated about taking my medication
☐ Yes, because of side effects or fear of having them
☐ Yes, because of complex regimens and/or inconvenient regimens
☐ Yes, for other reasons

[a] The Haynes-Sackett questionnaire score is based on the response to item 1: No (adherent) or Yes (non-adherent).
[b] The 6 additional responses were added to improve provider understanding of patients' difficulties with medication for use with the mHeart platform. The patient is able to tick more than one response.
