# Supplementary material for: A Mobile App (mHeart) to Detect Medication Nonadherence in the Heart Transplant Population: Validation Study
Source: JMIR Mhealth Uhealth. 2020 Feb 4;8(2):e15957. doi: 10.2196/15957 (PMC7055830; doi:10.2196/15957)
Supplement: Multimedia Appendix 6 [file mhealth_v8i2e15957_app6.pdf]

This is a Multimedia Appendix to a full manuscript published in the J Med Internet Res.
For full copyright and citation information see http://dx.doi.org/10.2196/jmir.xxxx

Multimedia Appendix 6. Patient Satisfaction and Usability Survey Original Spanish Version (the results and items translation to English language are provided in Multimedia Appendix 11).

Encuesta de Satisfacción de la Plataforma Nabelia Salud - Trasplante Cardíaco. Hospital de la Santa Creu i Sant Pau

* Obligatòria

¡Bienvenido!

El equipo de Trasplante Cardíaco del Hospital de la Santa Creu i Sant Pau, le da las gracias por responder las preguntas que le adjuntamos a continuación.

Su opinión sobre la plataforma Nabelia Trasplante cardíaco es muy importante para nosotros.

Si tiene cualquier duda llámenos al 661787173.

1. **Antes de empezar, ¿nos puede indicar cuánto tiempo (en años) hace del trasplante?**

   ____

2. **La frecuencia con la que utilizo la aplicación móvil o página web es:** *

   Maqueu només un oval.

   - ( ) No la uso
   - ( ) Ocasionalmente
   - ( ) Cada 15 días
   - ( ) Cada semana
   - ( ) Dos o tres veces por semana
   - ( ) Cada día

3. **Habitualmente utilizo la plataforma a través de:** *

   Maqueu només un oval.

   - ( ) Página web
   - ( ) Aplicación móvil (app)
   - ( ) Ambas por igual

4. **El uso de la plataforma y su funcionamiento general me resulta:** *

Conteste un número del 1 al 10 (1.Muy complicado - 10.Muy sencillo)
Maqueu només un oval.

- 1 . Muy complicado
- 2
- 3
- 4
- 5
- 6
- 7
- 8
- 9
- 10. Muy sencillo
- No sabe/No contesta

5. **El usuario y la contraseña para acceder a la plataforma me resultan simples y fáciles de recordar:** *

Conteste un número del 1 al 10 (1.Poco - 10.Mucho)
Maqueu només un oval.

- 1 . Poco
- 2
- 3
- 4
- 5
- 6
- 7
- 8
- 9
- 10. Mucho
- No sabe/No contesta

6. **La formación inicial telefónica que recibí de Nabelia sobre el uso de la plataforma, me resultó:** *

Conteste un número del 1 al 10 (1.Poco útil - 10.Muy útil)
Maqueu només un oval.

- 1 . Poco útil
- 2
- 3
- 4
- 5
- 6
- 7
- 8
- 9
- 10. Muy útil
- No he recibido formación en el uso de la plataforma

7. **Si he tenido dudas y/o incidencias, el soporte técnico de Nabelia me ha resultado:** *

Conteste un número del 1 al 10 (1.Poco útil - 10.Muy útil)
Maqueu només un oval.

- 1 . Poco útil
- 2
- 3
- 4
- 5
- 6
- 7
- 8
- 9
- 10. Muy útil
- No he tenido dudas/incidencias

8. **Anotar el peso, tensión, frecuencia cardíaca, etc. en la plataforma, me incentiva a llevar un mejor control:** *

Conteste un número del 1 al 10 (1.Poco - 10.Mucho)
Maqueu només un oval.

- 1 . Poco
- 2
- 3
- 4
- 5
- 6
- 7
- 8
- 9
- 10. Mucho
- No sabe/No contesta

9. **Registrar el peso, tensión, frecuencia cardíaca, etc. en la plataforma, me resulta:** *

Conteste un número del 1 al 10 (1.Complicado - 10. Sencillo)
Maqueu només un oval.

- 1 . Complicado
- 2
- 3
- 4
- 5
- 6
- 7
- 8
- 9
- 10. Sencillo
- No sabe/No contesta

10. **Los avisos en la agenda para recordar que registre la tensión, peso, frecuencia cardíaca, etc., me resultan:** *

Conteste un número del 1 al 10 (1.Molesta - 10. Conveniente)
Maqueu només un oval.

- 1 . Molesta
- 2
- 3
- 4
- 5
- 6
- 7
- 8
- 9
- 10. Conveniente
- No sabe/No contesta

11. **Si tengo algún síntoma (cefalea, diarrea, dolor muscular, etc.), lo registro en el apartado de síntomas de la plataforma:** *

Conteste un número del 1 al 10 (1. Nunca - 10. Siempre)
Maqueu només un oval.

- 1 . Nunca
- 2
- 3
- 4
- 5
- 6
- 7
- 8
- 9
- 10. Siempre
- No he tenido síntomas que registrar

12. **El registro de los síntomas en la plataforma me resulta:** *

Conteste un número del 1 al 10 (1. Complicado - 10. Sencillo)
Maqueu només un oval.

- 1 . Complicado
- 2
- 3
- 4
- 5
- 6
- 7
- 8
- 9
- 10. Sencillo
- No sabe/No contesta

13. **El apartado de Consejos de la plataforma me resulta:** *

Conteste un número del 1 al 10 (1.Poco útil - 10.Muy útil)
Maqueu només un oval.

- 1 . Poco útil
- 2
- 3
- 4
- 5
- 6
- 7
- 8
- 9
- 10. Muy útil
- No sabe/No contesta

14. **He consultado los consejos a través de la plataforma:** *

Maqueu només un oval.

- Nunca los he consultado
- Entre 1 y 5 veces
- Más de 5 veces

15. **¿Qué información le gustaría que apareciera en los Consejos?** *

Se puede marcar más de una opción. Puede añadir sugerencias en el campo libre.
Seleccioneu totes les opcions que corresponguin.

- [ ] Videos informativos realizados por el personal sanitario que le atiende
- [ ] Páginas web de consulta sobre trasplante
- [ ] Páginas web de consulta sobre su salud en general
- [ ] Altres: ____________________

16. **Para consultar si puedo tomar una nueva terapia (medicamento, plantas, infusiones, homeopatía u otra terapia alternativa), utilizo:** *

Se puede marcar más de una opción.
Seleccioneu totes les opcions que corresponguin.

- [ ] El apartado de "nuevo tratamiento" de la plataforma
- [ ] La mensajería de la plataforma
- [ ] El teléfono, llamo a la farmacéutica
- [ ] El teléfono, llamo al médico
- [ ] No he tenido que consultar
- [ ] No sabe/No contesta

17. **Registrar en la agenda si tomo los medicamentos, me resulta:** *

Conteste un número del 1 al 10 (1. Molesto - 10. Conveniente)
Maqueu només un oval.

- 1 . Molesto
- 2
- 3
- 4
- 5
- 6
- 7
- 8
- 9
- 10. Conveniente
- No sabe/No contesta

18. **Recibir avisos con los cambios de tratamiento, me resulta:** *

Conteste un número del 1 al 10 (1.Poco útil - 10.Muy útil)
Maqueu només un oval.

- 1 . Poco útil
- 2
- 3
- 4
- 5
- 6
- 7
- 8
- 9
- 10. Muy útil
- No sabe/No contesta

19. **Recibir recordatorios en mi móvil del horario de las tomas de la medicación, me resulta:** *

Conteste un número del 1 al 10 (1.Molesto - 10. Conveniente)
Maqueu només un oval.

- 1 . Molesto
- 2
- 3
- 4
- 5
- 6
- 7
- 8
- 9
- 10. Conveniente
- No sabe/No contesta

20. **Utilizo la agenda de la plataforma como agenda personal:** *

Conteste un número del 1 al 10 (1. Nunca - 10. Siempre)
Maqueu només un oval.

- 1 . Nunca
- 2
- 3
- 4
- 5
- 6
- 7
- 8
- 9
- 10. Siempre
- No sabe/No contesta

21. **Los cuestionarios de cumplimiento y actitud frente a la terapia me resultan:** *

Conteste un número del 1 al 10 (1. Molestos - 10. Adecuados)
Maqueu només un oval.

- 1 . Molestos
- 2
- 3
- 4
- 5
- 6
- 7
- 8
- 9
- 10. Adecuados
- No sabe/No contesta

22. **Utilizar la mensajería de la plataforma para contactar con mi profesional sanitario, me resulta:** *
Conteste un número del 1 al 10 (1. Complicado de utilizar - 10. Fácil de utilizar)
Maqueu només un oval.

- 1 . Complicado de utilizar
- 2
- 3
- 4
- 5
- 6
- 7
- 8
- 9
- 10. Fácil de utilizar
- No sabe/No contesta

23. **Disponer de la mensajería de la plataforma para resolver dudas con mi farmacéutica o enfermera, me resulta:** *
Conteste un número del 1 al 10 (1.Poco útil - 10.Muy útil)
Maqueu només un oval.

- 1 . Poco útil
- 2
- 3
- 4
- 5
- 6
- 7
- 8
- 9
- 10. Muy útil
- No sabe/No contesta

Passeu a la pregunta 24.

Para finalizar, queremos saber su opinión sobre qué le está aportando la plataforma que hemos desarrollado para usted.

La finalidad del equipo de trasplante, es adaptar el uso de la plataforma a cada persona.

Con sus respuestas nos ayuda a mejorar.

24. **Desde que uso la plataforma, me siento:** *

Puede marcar más de una opción.
Seleccioneu totes les opcions que corresponguin.

- [ ] Acompañado por mi equipo asistencial
- [ ] Más seguro al poder consultar mis dudas
- [ ] Que controlo mejor mi salud en general
- [ ] Sobrecargado por las tareas en la plataforma
- [ ] Abrumado por los mensajes de mi equipo asistencial
- [ ] Altres: ______________________

25. **¿Tiene alguna sugerencia de mejora de la plataforma?** *

Seleccioneu totes les opcions que corresponguin.

- [ ] No, me gusta tal y como está
- [ ] Altres: ______________________

26. **¿Recomendaría el uso de la plataforma a otras personas trasplantadas?** *

Maqueu només un oval.

- ( ) Sí, recomendaría su uso *Passeu a ¡Muchas gracias!.*
- ( ) No recomendaría su uso *Passeu a la pregunta 27.*

Passeu a ¡Muchas gracias!.

27. **Le agradecemos su sinceridad, indique el motivo por el cual no recomendaría el uso de la plataforma:** *

____
____
____
____
____

¡Muchas gracias!

Su opinión nos resulta imprescindible para mejorar el aplicativo que hemos diseñado para usted.

El equipo clínico del Hospital de la Santa Creu i Sant Pau le agradece su colaboración.

Tecnologia de Google Forms
