# Supplementary material for: A Mobile App (mHeart) to Detect Medication Nonadherence in the Heart Transplant Population: Validation Study
Source: JMIR Mhealth Uhealth. 2020 Feb 4;8(2):e15957. doi: 10.2196/15957 (PMC7055830; doi:10.2196/15957)
Supplement: Multimedia Appendix 7 [file mhealth_v8i2e15957_app7.pdf]

This is a Multimedia Appendix to a full manuscript published in the J Med Internet Res.
For full copyright and citation information see http://dx.doi.org/10.2196/jmir.xxxx

Multimedia Appendix 7. Methodology used to measure validity properties of the electronic PROMs to assess medication adherence in the at-home setting using the mHeart platform in heart transplant recipients (The Val-mHeart Study)

Definition of the measurement property[a]	Statistical analysis[a]	Study method
**Content validity**<br>The degree to which a PROM includes the most relevant aspects of a concept in the context of a given measurement application[1].		
The validity in this study begins with the adequate representability and relevance of the questionnaires selected by the experts to measure medication adherence[b] by the mHeart platform.	The inter-rater agreement between the expert panel[2,3] was calculated based on the Group Consensus Method. [4]<br><br>Agreement measurement: (1) percentages of agreement. Agreement among >75% of the expert panel was considered adequate.<br>(2) *P*-value on-side to test if *P* was greater than 0.75% – 75%. A greater *Proportion* implied greater agreement between experts.	The expert panel consisted of 14 health professionals including 3 nurses, 7 cardiologists and 1 pharmacist of the heart failure and heart transplant unit, as well as 1 pharmacist specialized in medication adherence and 2 clinical pharmacists with experience in in-clinic motivational interviews.<br>The pharmacists proposed 3 medication adherence PROMs broadly used in in-clinic practice: the Haynes-Sackett,[5] Morisky-Green-Levine[6] and the SMAQ[7] questionnaires. The selection of 2 of them for implementation in the mHeart tool had to be based on: adequacy of the PROMs to be performed through an app, validation and experience of use of the PROMs, and the degree to which the instruments measure complementary constructs of medication adherence.<br>The experts were asked to evaluate these criteria and whether the suggested PROMs were adequate for implementation in the mHeart platform. The discussion was verbal and panel members voted by rising their hands.
The degree to which the new 6 difficulties with medication implemented in the Haynes-Sacket ePROM (Multimedia Appendix 2) are an adequate reflection of the construct to be measured.	The inter-rater agreement among the expert panel[2,3] was calculated based on the Nominal group consensus method[4].<br>Same as above.	The same expert panel was asked to evaluate by written record if they agreed (Yes/No) with different criteria for each of the 6 medication difficulties of the Haynes-Sacket ePROMs. Afterward, a moderated verbal discussion took place.<br>• Useful to evaluate medication adherence<br>• Intuitive<br>• Brief (can be completed rapidly)<br>• Patient-friendly language
The degree to which the items of the ePROM itself are an adequate reflection of the construct to be measured compared with the original in-clinic PROM.	The inter-rater agreement among the expert panel [2,3] was calculated based on the Nominal group consensus method. [4]<br>Agreement measurement: Kappa coefficient calculated for the overall PROM score and for each item.	The same expert panel was asked to evaluate by written record if they agreed (Yes/No) with several characteristics of the electronic version compared with the in-clinic PROM. Then, a moderated verbal discussion took place.<br>• True to the original text<br>• Useful to evaluate medication adherence<br>• Intuitive<br>• Brief (can be completed rapidly)<br>• Patient-friendly language
**Convergent validity**		
The degree to which the Morisky-Green-Levine PROM score is consistent with other instruments based on the assumption that measures the same construct.	Positive correlation with another instrument measuring the same construct.<br>Correlation measurement: Phi coefficient.	To assess the correlation of the scores between the Morisky-Green-Levine PRO, both electronic and in-clinic, with the in-clinic SMAQ[7] instrument. The SMAQ questionnaire was adapted from the Morisky-Green-Levine and validated in the transplant population as an adequate reflection of the dimensionality of the construct of adherence to be measured.
**Discriminant validity (divergent validity)**		
The degree to which the Haynes-Sacket PROM score is not consistent with other instruments based on the assumption that this PROM measures another construct.	Negative correlation or lack of correlation between PROMs measuring different constructs.<br>Correlation measurement: Phi coefficient.	To assess the differences in the scores between the ePROMs implemented in the platform (Haynes-Sacket vs Morisky-Green-Levine), since the information provided by the 2 instruments are different and complementary.<br>The same comparison was made using the SMAQ PROM, which measures different adherent constructs of the Haynes-Sacket construct of adherence.
**Reliability or reproducibility**<br>The degree to which a PRO instrument is free from measurement error. [1,8]		
The degree to which the 2 versions of the same test (the electronic and in-clinic adherence PROMs) measure the same construct.	Equivalent forms reliability method. [2]<br>Measure of association: Phi coefficient (reliability coefficient).	To ask to the same group of patients and on the exact same day to independently perform the in-clinic PROMs and the mHeart ePROMs.<br>To assess the association between score versions of the electronic PROMs or the in-clinic PROMs.
The extent to which the scores of the test are consistent for repeated	Test-retest reliability method. [2]	To ask patients to complete the ePROMs 7 days after the last completion. The patients must remain clinically and therapeutically stable during this 7-day period to be included in the test-retest analysis.

measurement over time in patients who have not changed (score stability measurement).	Agreement measurement: Kappa coefficient [9] (stability or reproducibility coefficient).	To compare the ePROMs scores obtained in Assessments 2 and 3.<br>The 7-day gap was selected to minimize the effect of possible confounding variables[9] related to the multifaceted factors affecting the patient's medication adherence post-transplant. [10] Intervals of 1-2 weeks appear to be typical in reproducibility of health status measures. [11]
**Responsiveness or sensitivity to change**		
The responsiveness or sensitivity to change is the ability of the PROM to detect change over time in the construct being measured.	Percentage of adherent and non-adherent recipients. Statistical test to calculate the change in significance of *P*-values.	To measure the medication adherence PROMs overall scores for each visit.<br>To calculate the improvement in medication adherence between assessments, as a measure of the intervention effect.<br>The follow-up time between visits was at least 4 weeks. The validity of an indirect measures is adequate if it includes 1-month periods. [12] Smartphone use cycles with a time gap of 4 weeks between them are highly likely to be independent cycles. [13]
**Interpretability**		
The degree to which easily understood meaning can be assigned to an instrument's score and the meaningful level of change.	1. Score interpretation.<br>2. Meaningful change.<br>3. Comparison of scores in the transplant population.	To respond to the following questions: are the scores easily interpreted? What does a high or low score represent? Is the baseline rate different or similar to that of other studies? What comprises a meaningful difference in the score compared with other series in transplant populations?
**Respondent burden**		
Burden is defined as the time, effort, and other demands placed on respondents who are administered the instrument.	The SAC-MOS guidelines[14] recommendations suggests various review criteria for respondent burden.	To analyze the following criteria with the ePROMs versus the in-clinic PROMs from the respondents' point of view:<br>1. Average and range of time needed to complete the instrument.<br>2. The level of comprehension needed for the population.<br>3. Indication of when or under what circumstances the instrument is not suitable for respondents.<br>4. The acceptability of the instrument by indicating the level of missing data and the reasons.<br>5. Provision of evidence that the instrument places no undue physical or emotional strain on respondents.
**Administrative burden**		
Burden is defined as the time, effort, and other demands placed on persons administering the instrument.	The SAC-MOS guidelines[14] suggest various review criteria for administrative burden.	To analyze the following criteria with the ePROMs versus the in-clinic PROMs from the administrative point of view:<br>1. Resources required for administration of the instrument.<br>2. Special requirements such as the need to record the results into the HIS or EHR.<br>3. The average time and range of time required by a trained interviewer to administer the instrument in face-to-face interviews or through the mHeart platform.<br>4. The amount of training needed, and level of education or professional expertise and experience needed to administer, score or use the instrument.<br>5. The availability of the scoring instructions for the PROMs.

[a] The definition of the properties, statistical analysis and methodology was adapted to this study based on the SAC-MOS Guidelines, the COSMIN consensus and the ISOQOL standards.

[b] Non-adherence to medication in the implementation phase was defined as "actual dosing does not correspond to the prescribed dosing regimen due to delays, omissions or extra doses" and was measured by self-report questionnaires. Delay refers to irregularities in the intake schedule (±2 hours).

EHR, health electronic records, ePROMs, electronic patient-reported outcome measures identified by the mHeart tool; HIS, hospital information system; in-clinic PROMs, patient-reported outcome measures identified by a face-to-face interview in the clinic; PROMs, patient-reported outcome measures.

References

1. Reeve BB, Wyrwich KW, Wu AW, Velikova G, Terwee CB, Snyder CF, et al. ISOQOL recommends minimum standards for patient-reported outcome measures used in patient-centered outcomes and comparative effectiveness research. *Qual Life Res*. 2013;22(8):1889-1905. PMID:23288613
2. Viladrich C, Doval E. *Medición: Fiabilidad y Validez. 5ª Ed Bellaterra: Laboratori d'estadística Aplicada i Modelització, Universitat Autónoma de Barcelona (UAB).*; 2011.
3. Cohen J. A coefficient of agreement for nominal scales. *Educ Psychol Meas*. 1960;20(1):37-46 ST-A coefficient of agreement for nominal. DOI:10.1177/001316446002000104
4. Isabel de Arquer M. *Fiabilidad Humana: Métodos de Cuantificación, Juicio de Expertos [Human Reliability: Quantification Methods, Experts Judgement].*; 1994:1-5.
5. Sackett DL, Snow JC. The magnitude of compliance and non-compliance. *Haynes RB Taylor DW, Sackett DL, Eds Compliance Heal Care Maryl Johns Hopkins Univ Press*. 1979:207-225.
6. Morisky DE, Green LW, Levine DM. Concurrent and predictive validity of a self-reported measure of medication adherence. *Med Care*. 1986;24(1):67-74. PMID:3945130
7. Ortega Suárez FJ, Sánchez Plumed J, Pérez Valentín MA, Pereira Palomo P, Muñoz Cepeda MA, Lorenzo Aguiar D, et al. Validation on the simplified medication adherence questionnaire (SMAQ) in renal transplant patients on tacrolimus. *Nefrologia*. 2011;31(6):690-696. PMID:22130285
8. Spearman C. Proof and measurement of association between two things. *Am J Psychol*. 1904;15(1):72-11.
9. Aaronson N, Alonso J, Burnam A, Lohr K, Patrick D, Perrin E, et al. Scientific Advisory Committee of the Medical Outcomes Trust. Assessing health status and quality-of-life instruments: Attributes and review criteria. *Qual Life Res*. 2002;11(3):193-205.

PMID:12074258

10. Kini V, Ho PM. Interventions to Improve Medication Adherence A Review. *JAMA*. 2018;320(23):2461-2473. PMID:30561486
11. Polit DF. Getting serious about test-retest reliability: A critique of retest research and some recommendations. *Qual Life Res*. 2014;23(6):1713-1720. PMID:24504622
12. Conthea P, Márquez Contreras E, Aliaga Pérezc A, Barragán Garcíad B, Fernández de Cano Martin MN, González Juradof M, et al. Adherencia terapéutica en la enfermedad crónica: estado de la situación y perspectiva de futuro. *Rev Clin Esp*. 2014;214(6):336-344. DOI:10.1016/j.rce.2014.03.008
13. Pan Y-C, Lin H-H, Chiu Y-C, Lin S-H, Lin Y-H. Temporal Stability of Smartphone Use Data: Determining Fundamental Time Unit and Independent Cycle (Preprint). *JMIR mHealth uHealth*. 2019;7:1-9. PMID:30912751
14. Aaronson N, Alonso J, Burnam A, Lohr KN, Patrick DL, Perrin E, et al. Assessing health status and quality-of-life instruments: attributes and review criteria. *Qual Life Res*. 2002;11(3):193-205. PMID:12074258
