# Supplementary material for: A Mobile App (mHeart) to Detect Medication Nonadherence in the Heart Transplant Population: Validation Study
Source: JMIR Mhealth Uhealth. 2020 Feb 4;8(2):e15957. doi: 10.2196/15957 (PMC7055830; doi:10.2196/15957)
Supplement: Multimedia Appendix 8 [file mhealth_v8i2e15957_app8.pdf]

This is a Multimedia Appendix to a full manuscript published in the J Med Internet Res.
For full copyright and citation information see http://dx.doi.org/10.2196/jmir.xxxx

Multimedia Appendix 8. Demographic and clinical characteristics of the early-stage heart transplant recipients included in the Val-mHeart Study

**Variables**	**N = 31**
Recipient gender (women), n (%)	9 (29)
Recipient age at the time of the study, years ± SD	54 ± 12
Donor age, years ± SD	49 ± 12
Donor sex (men), n (%)	13 (42)
Time from HTx transplant, years ± SD	1.2 ± 0.8
Body mass index, kg/m² ± SD	25 ± 54
Cytomegalovirus mismatch (recipient-/donor+), n (%)	5 (16)
Heart failure etiology, n (%)	
• Congenital	4 (13)
• Coronary/ischemic	12 (39)
• Myocarditis	1 (3.2)
• Cardiomyopathy	12 (39)
• Hypertrophic cardiomyopathy	1 (3)
• Other	1 (3)
Urgent HTx, n (%)	15 (48)
Total ischemia time, min±SD	198 ± 48
Number of recipients with at least 1 episode of ARE, median (Q1-Q3)	4 (13)
AMR, n (%)	1 (5)
CAV, n (%)	9 (29)
LVEF, %±SD	66 ± 8
Educational attainment, n (%)	
• No schooling	5 (16)
• Middle school graduate	13 (42)
• High school graduate	7 (23)
• University graduate	6 (19)
Employment status, n (%)	
• Temporary medical leave	8 (26)
• Long-term Disability	12 (39)
• Retired	7 (23)
• No previous employment	2 (7)
• Currently employed	2 (7)
Need or requirement for caregiver, n (%)	11 (36)
Lives with someone else, n (%)	28 (90)
Number of comorbidities	
• pre-transplant, mean ± SD (range)	3.4 ± 2.5
• post-transplant, mean ± SD (range)	2.8 ± 2.3
Medical clinicians (other than the transplant team), mean±SD (range)	2.4 ± 0.8
Number of patients who were always visited by the same primary care physician, n (%)	28 (90)
Number of primary care visits in the last month, n (%)	
• None	20 (65)
• 1-2 visits	11 (36)
• >3 visits	None
Reasons for primary care visits, n (%)	
• Medical consultation	8 (26)
• Refill prescriptions	20 (65)
• Other	3 (10)
mHeart initial patient assessment; n (%)	
• Not very useful	4 (13)
• Useful	22 (71)
• Very useful	4 (13)
• Not yet known until the platform is tested	1 (3)
Frequency of technology use; n (%)	
• Frequently	22 (71)
• Occasionally	6 (19)
• Never	3 (10)
Use of health-related technology	22 (71)
Patient assistance in using the platform, n (%)	
• Yes	9 (29)
• No	22 (71)
• Not yet known until the platform is tested	0 (0)

AMR, antibody-mediated rejection; ARE, acute cellular rejection episode; BMI, body mass index; CAV, cardiac allograft vasculopathy; HTx, heart transplantation; LVEF, left ventricular ejection fraction; SD, standard deviation.
