# Supplementary material for: A Mobile App (mHeart) to Detect Medication Nonadherence in the Heart Transplant Population: Validation Study
Source: JMIR Mhealth Uhealth. 2020 Feb 4;8(2):e15957. doi: 10.2196/15957 (PMC7055830; doi:10.2196/15957)
Supplement: Multimedia Appendix 9 [file mhealth_v8i2e15957_app9.pdf]

This is a Multimedia Appendix to a full manuscript published in the J Med Internet Res.
For full copyright and citation information see http://dx.doi.org/10.2196/jmir.xxxx

Multimedia Appendix 9. Heart transplant recipients' therapeutic characteristics and treatment-related patient-reported outcomes (PRO)

Variables	N = 31
**Total number of drugs prescribed**, mean ± SD (range)	13 ± 4 (7-18)
**Patients with polypharmacy**, n (%)	
• ≥ 5 drugs; ≥ 8 drugs; ≥ 14 drugs	31 (100); 27 (87); 11 (34)
**Medication categories**, n (%)	
• Immunosuppressants, mean ± SD (range)	2.9 ± 0.4 (2-4)
○ mycophenolate mofetil	22 (71)
○ mycophenolate sodium	6 (19)
○ azathioprine	0
○ cyclosporine	1 (3)
○ tacrolimus extended release	14 (45)
○ tacrolimus immediate release	16 (52)
○ sirolimus	0
○ everolimus	2 (6)
○ prednisone	30 (97)
• Other treatments established in HTx protocol	
○ calcium + vitamin D	29 (94)
○ Aspirin	23 (74)
○ pravastatin	26 (84)
○ valganciclovir	16 (52)
○ cotrimoxazole + folinate acid	18 (58)
○ antacid (IBP or antiH2)	30 (97)
• Other drugs to treat comorbidities > 20% (ATC code)	
○ Group A. Alimentary tract and metabolism	25 (20)
○ Group C. Cardiovascular system	23 (19)
○ Group G. Genitourinary system and sex hormones	17 (14)
○ Group N. Nervous system	32 (26)
• Over-the-counter drugs (OTC), mean ± SD (range)	2 ± 1
**Patient autonomous for preparing and taking medication**, n (%)	19 (61)
**Reasons for lack of autonomy in medication intake**, n (%)	
• Reports neurological symptoms limiting ability (eg, confusion, lack of memory)	4 (33)
• Reports fear of forgetting unless given help	1 (8)
• Reports receiving a lot of information after transplantation	3 (25)
• Does not know the reason	4 (33)
**Knowledge of the therapeutic regime**	
• Names of the drugs remembered	
○ Mean (range)	6 (0-16)
○ Proportion of drugs remembered the total prescribed, %	54
• Doses of the drugs remembered	
○ Mean (range)	2 (0-7)
○ Proportion of drugs remembered of the total prescribed, %	25
• Intake of the drugs remembered	
○ Mean (range)	6 (0-13)
○ Proportion of drugs remembered of the total prescribed, %	59
• Indications of the drugs remembered	
○ Mean (range)	5 (0-13)
○ Proportion of drugs remembered of the total prescribed, %	43
**Degree of inconvenience perceived by patients related to taking their medication as prescribed every day (scale 0-10)**, mean ± SD (P 25, 50, 75, 90)	4 ± 3 (2, 3, 6, 7)
**Patients' perception of taking excessive medication**, n (%)	23 (74)
**Patients' awareness of the importance of immunosuppressive therapy,** n (%)	
1. If you discontinued taking your immunosuppressants completely, what do you think would happen to you?	
• Nothing	1 (3)
• I don't know	10 (32)
• A different answer involving rejection	20 (65)
2. If you sometimes forgot to take your immunosuppressants, what do you think would happen to you?	
• Nothing	1 (3)
• I don't know	13 (42)
• A different answer involving rejection	17 (55)
3. Did you modify the immunosuppressant timetable in the last week?	
• No	23 (74)
• > once	8 (26)

This is a Multimedia Appendix to a full manuscript published in the J Med Internet Res.
For full copyright and citation information see http://dx.doi.org/10.2196/jmir.xxxx

• I don't remember	0 (0)
4. Did you modify the immunosuppressant timetable since the last visit?	
• No	21 (68)
• > once	5 (16)
• >5 times	5 (16)
• I don´t remember	0 (0)
**Patient's reported adverse effects**, mean ± SD (range)	6 ± 4 (0-11)
• ≥ 2 adverse effects	25 (81)
• ≥ 5 adverse effects	19 (61)
**Type of adverse effect reported by patients**, n (%)	
• Visual impairment	43 (25)
• Psychological (emotional disorders, insomnia)	42 (24)
• Neurological (tremor, dizziness, headache)	39 (22)
• Mucosa and aesthetic (thrush, gingival disorder alopecia, increased hair growth, other skin disorders and visual disorders)	22 (13)
• Pain (muscular pain, joint pain)	15 (9)
• Gastric (diarrhea, nausea, constipation, vomiting, etc.)	12 (7)
• Muscular (weariness, tiredness or fatigue, cramps)	2 (1)

HTx, heart transplant; SD, standard deviation.
