# Supplementary material for: A Mobile App (mHeart) to Detect Medication Nonadherence in the Heart Transplant Population: Validation Study
Source: JMIR Mhealth Uhealth. 2020 Feb 4;8(2):e15957. doi: 10.2196/15957 (PMC7055830; doi:10.2196/15957)
Supplement: Multimedia Appendix 10 [file mhealth_v8i2e15957_app10.pdf]

This is a Multimedia Appendix to a full manuscript published in the J Med Internet Res.
For full copyright and citation information see http://dx.doi.org/10.2196/jmir.xxxx

Multimedia Appendix 10. Non-adherence rates in early-stage heart transplant recipients listed by item in the study period

N=31	Assessment 1		Assessment 2		Assessment 3
**Measure**	**In-clinic**	**mHeart**	**In-clinic**	**mHeart**	**mHeart**
**Haynes-Sackett**					
• Overall score	29%	19%	10%	7%	0%
• HS 1	NA	7%	NA	3%	0%
• HS 2	NA	0%	NA	0%	0%
• HS 3	NA	0%	NA	0%	0%
• HS 4	NA	3%	NA	0%	0%
• HS 5	NA	3%	NA	0%	0%
• HS 6	NA	7%	NA	3%	0%
**Morisky-Green-Levine**					
• Overall score	32%	19%	13%	10%	3%
• MGL 1	26%	16%	3%	3%	0%
• MGL 2	0%	3%	0%	0%	0%
• MGL 3	7%	10%	4%	7%	0%
• MGL 4	1%	3%	1%	0%	0%
**SMAQ**					
• Overall score	39%	NA	13%	NA	NA
• SMAQ 1	26%	NA	3%	NA	NA
• SMAQ 2	0%	NA	0%	NA	NA
• SMAQ 3	23%	NA	4%	NA	NA
• SMAQ 4	3%	NA	0%	NA	NA
• SMAQ 5					
1-2 days	7%	NA	2%	NA	NA
>3 days	3%	NA	0%	NA	NA
• SMAQ 6		NA		NA	NA
1 day	19%	NA	7%	NA	NA
≥ 2 days	7%	NA	3%	NA	NA

[a] Non-adherence to medications refers to the implementation phase, is defined as “actual dosing does not correspond to the prescribed dosing regimen due to delays, omissions or extra doses” and is measured by self-report questionnaires. Delays refer to irregularities with the intake schedule (±2 hours).

ePROMs, electronic patient-reported measures; NA, not applicable.
