# Supplementary material for: A Mobile App (mHeart) to Detect Medication Nonadherence in the Heart Transplant Population: Validation Study
Source: JMIR Mhealth Uhealth. 2020 Feb 4;8(2):e15957. doi: 10.2196/15957 (PMC7055830; doi:10.2196/15957)
Supplement: Multimedia Appendix 11 [file mhealth_v8i2e15957_app11.pdf]

This is a Multimedia Appendix to a full manuscript published in the J Med Internet Res.
For full copyright and citation information see http://dx.doi.org/10.2196/jmir.xxxx

Multimedia Appendix 11. Results of the online survey on usability and satisfaction with the mHeart platform and intervention implemented during the study period in heart transplant recipients. Table 11A shows the categorical variables and table 11B the quantitative variables

**Table 11A.** **Categorical variables, n (%)**	**N = 29**
**I usually use the platform through:**	
• Mobile application (app)	26 (90)
• Webpage	2 (7)
• Both equally	1 (3)
**My frequency of use of the mHeart mobile app is:**	
• I don´t use it	0 (0)
• Occasionally	2 (7)
• Every 15 days	1 (4)
• Every week	4 (14)
• 2 or 3 times a week	3 (10)
• Every day	19 (66)
**I´ve looked the Health Advice Module through the platform:**	
• Never	5 (17)
• Between 1 and 5 times	18 (62)
• More than 5 times	6 (21)
**What information would you like to see in the Health Advice Module:** (more than 1 answer is allowed)	
• Information videos made by heart transplant staff	19 (66)
• Medical advice website on transplants	8 (28)
• Medical advice website on health in general	8 (28)
• Transplant protocols	1 (3)
• Other	4 (14)
• Don´t know/no answer	3 (10)
**To find out whether I can take a new therapy (drug, herbal, infusions, homeopathy, etc.) I use:** (more than 1 answer is allowed)	
• The "new treatment" feature of the platform	6 (21)
• The platform's chat system	19 (66)
• Telephone, I ring the pharmacist	15 (52)
• Telephone, I ring the doctor	0 (0)
• I haven´t had to make any enquiries	3 (10)
• I don´t know/no answer	0 (0)
**Since I´ve been using the platform, I feel:** (more than 1 answer is allowed)	
• Supported by my healthcare team	18 (62)
• More secure because I can clear up my doubts	23 (79)
• More in control of my health in general	9 (31)
• Overloaded by the tasks in the platform	1 (3)
• Overwhelmed by the messages from my healthcare team	0 (0)
• Other (free field)	0 (0)
**Would you recommend using the platform to other transplant recipients?**	
• Yes, I´d recommend its use	29 (100)
• No, I wouldn't recommend its use	0 (0)
○ Thank you for your honesty. Please say why you wouldn´t recommend using the platform	-
**Do you have any suggestions for improving the platform?**	
• No, I like it just as it is	22 (76)
• **Other (free field):**	7 (24)
Patient 1: *"Some things should be improved"*	-
Patient 2: *The questions on the attitude to medication are always the same and in the same order. This makes us not pay attention when we've given more than two responses. I think the order should be mixed up.*	Improved
Patient 3: *Problems with messages and alerts about intakes in the mobile app make the app not useful.*	Improved
Patient 4: *Registration of medication intake could be per day or like now.*	Explained to the patient
Patient 5. (A) *To record blood pressure, you first have to delete the numbers that appear and sometimes it´s quite difficult because the keys and the cursor arrow appear on the screen at the same time.*	Improved
Patient 5. (B) *What can you do if you realize you've made a mistake in a number that you've already sent?*	Explained to the patient
Patient 6: *It would be better to send alerts of a new message, without the patient having to go to the update button.*	Improved
Patient 7: *I´d like to see the graphs on my mobile.*	Improved

**Table 11B.** **Quantitative variables (N=29), n (%)**	**1**	**2**	**3**	**4**	**5**	**6**	**7**	**8**	**9**	**10**	**DN/NA**	**Mean ± SD (0 to 10 score)**
**I find the use of the platform and its general functioning:** 1. Very difficult 10. Very simple	0 (0)	0 (0)	0 (0)	0 (0)	2 (7)	2 (7)	1 (4)	8 (28)	3 (10)	13 (45)	0 (0)	**9 ± 2**
**The username and password to access the platform are simple and easy to remember:** 1. A little– 10. A lot	6 (21)	0 (0)	0 (0)	1 (4)	3 (10)	1 (4)	2 (7)	2 (7)	3 (10)	11 (38)	0 (0)	**7 ± 4**
**The initial telephone training I received from mHeart on the use of the platform was:** 1. Not very useful 10. Very useful	0 (0)	0 (0)	1 (4)	1 (4)	1 (4)	4 (14)	3 (10)	4 (14)	4 (14)	10 (35)	1 (4)	**8 ± 2**
**When I´ve had doubts and/or incidents, mHeart ‘s Help Center has been:** 1. Not very useful 10. Very useful	0 (0)	0 (0)	0 (0)	2 (7)	1 (4)	2 (7)	4 (14)	5 (17)	2 (7)	12 (41)	1 (4)	**8 ± 2**
**When I record my weight, blood pressure, heart rate, etc., on the platform, it motivates me to look after my health:** 1. A little 10. A lot	0 (0)	0 (0)	0 (0)	0 (0)	2 (7)	2 (7)	3 (10)	3 (10)	3 (10)	15 (52)	1 (4)	**9 ± 2**
**Recording my weight, blood pressure, heart rate, etc. on the platform is:** 1. Difficult 10. Simple	0 (0)	0 (0)	0 (0)	0 (0)	0 (0)	0 (0)	3 (10)	5 (17)	2 (7)	18 (62)	1 (4)	**9 ± 1**
**The alerts in the Agenda Module to remind me to record my blood pressure, weight, heart rate, etc., are:** 1. Annoying 10. Helpful	0 (0)	0 (0)	1 (4)	1 (4)	0 (0)	2 (7)	2 (7)	6 (21)	5 (17)	11 (38)	1 (4)	**8 ± 2**
**If I have a side effect, I record it in the Symptoms Module of the platform:** 1. Never 10. Always	6 (21)	1 (4)	2 (7)	4 (14)	1 (4)	2 (7)	2 (7)	2 (7)	1 (4)	5 (17)	3 (10)	**5 ± 3**
**Registering my symptoms in the platform is:** 1. Difficult 10. Easy	1 (4)	0 (0)	1 (4)	1 (4)	2 (7)	0 (0)	4 (14)	3 (10)	2 (7)	12 (41)	3 (10)	**8 ± 2.5**
**The health Advice Module of the platform is:** 1. Not very useful 10. Very useful	0 (0)	0 (0)	1 (4)	0 (0)	4 (14)	4 (13)	3 (10)	5 (17)	0 (0)	9 (31)	3 (10)	**8 ± 2**
**Registering whether I take my medication in the Agenda Module is:** 1. Annoying 10. Helpful	0 (0)	1 (4)	0 (0)	0 (0)	2 (7)	3 (10)	3 (10)	3 (10)	4 (14)	13 (45)	0 (0)	**8 ± 2**
**Receiving alerts with treatment changes is:** 1. Not very useful 10. Very useful	0 (0)	0 (0)	0 (0)	0 (0)	2 (7)	0 (0)	1 (4)	0 (0)	3 (10)	23 (79)	0 (0)	**9 ± 1**
**Receiving reminds in my mobile of when to take my medication is:** 1. Annoying 10. Helpful	0 (0)	0 (0)	1 (4)	0 (0)	1 (4)	3 (10)	1 (4)	0 (0)	4 (14)	18 (62)	1 (4)	**9 ± 2**
**The questionnaires on adherence and attitude to therapy are:** 1. Annoying 10. Appropriate	0 (0)	0 (0)	1 (4)	1 (4)	1 (4)	3 (10)	4 (14)	6 (21)	1 (4)	12 (41)	0 (0)	**8 ± 2**
**The platform’s Communication Module to contact my health professional is:** 1. Difficult to use – 10. Easy to use	0 (0)	0 (0)	0 (0)	0 (0)	2 (7)	1 (4)	1 (4)	3 (10)	1 (4)	(21) 72	0 (0)	**9 ± 2**
**Having the platform’s chat to resolve doubts with my pharmacist is:** 1. Not very useful 10. Very useful	0 (0)	0 (0)	0 (0)	0 (0)	1 (4)	0 (0)	2 (7)	1 (4)	2 (7)	23 (79)	0 (0)	**10 ± 1**

DN/NA, doesn´t know/no answer
